# Effects of Immediate and Delayed Repeated Cold Exposure After Physical Exertion: A Randomized Controlled Trial

**DOI:** 10.3390/ijerph23070887

**Published:** 2026-07-09

**Authors:** Callum Buehler, Ronja Romitti, Vanessa Wellauer, Ron Clijsen, Erich Hohenauer

**Affiliations:** 1 Rehabilitation and Exercise Science Laboratory (RESlab), Department of Business Economics, Health and Social Care, University of Applied Sciences and Arts of Southern Switzerland, 7302 Landquart, Switzerland; callum.buehler@supsi.ch (C.B.); ronja.romitti@supsi.ch (R.R.); vanessa.wellauer@supsi.ch (V.W.); ron.clijsen@supsi.ch (R.C.); 2 Department of Physiotherapy, International University of Applied Sciences THIM, 7302 Landquart, Switzerland; 3 School of Health Professions, Division of Physiotherapy, Bern University of Applied Sciences, 3012 Bern, Switzerland; 4 Department of Movement and Sport Sciences, Faculty of Physical Education and Physiotherapy, Vrije Universiteit Brussel, 1050 Brussels, Belgium; 5 Faculty of Sport Sciences, University of Poitiers, 86000 Poitiers, France; 6 Department of Movement and Sport Sciences, Faculty of Medicine, University of Fribourg, 1700 Fribourg, Switzerland

**Keywords:** cryotherapy, cold exposure, delayed-onset muscle soreness, maximal voluntary isometric contraction, eccentric exercise, recovery, randomized controlled trial, cold pack, inflammation, muscle thickness

## Abstract

**Highlights:**

**Public health relevance: How does this work relate to a public health issue?**

- Localized cryotherapy with reusable gel packs is one of the most widely used recovery and pain-management modalities in physiotherapy, rehabilitation, and community sports settings.
- Despite its near-universal use, evidence regarding the optimal timing of repeated local cold exposure remains inconsistent, leaving practitioners and the general public without clear guidance for everyday musculoskeletal care.

**Public health significance: Why is this work of significance to public health?**

- This randomized controlled trial directly compared immediate versus delayed repeated cold-pack application after exercise-induced muscle soreness, addressing a practice-versus-evidence gap that is relevant to large active populations.
- Neither immediate nor delayed local cooling produced a meaningful recovery advantage over a non-cooled control for soreness, strength, inflammatory markers, or muscle thickness, suggesting that the population-level benefit of self-administered local cold packs may be limited to subjective comfort.

**Public health implications: What are the key implications or messages for practitioners, policy makers and/or researchers in public health?**

- Practitioners can recommend local cold packs as a safe, well-tolerated and inexpensive comfort measure after intensive exercise but should not expect them to accelerate objective recovery.
- Policy makers and researchers should redirect attention towards cooling modalities with greater thermal reach (e.g., cold-water immersion or controlled cryotherapy systems) and towards studies that quantify intramuscular temperature, perfusion, and individual moderators such as subcutaneous fat thickness.

**Abstract:**

Background: Local cryotherapy is widely used to support recovery after exercise-induced muscle damage, but the optimal timing of cold application remains unclear. This study evaluated whether immediate or delayed local cooling influences recovery from exercise-induced muscle fatigue. Methods: Forty-eight healthy adults (39 women, 9 men; age 22 ± 3 years) performed a single-leg eccentric leg-extension protocol to induce delayed-onset muscle soreness (DOMS) and were randomized to immediate cooling (ICG), delayed cooling (DCG; starting 24 h post-exercise), or control (CG) in a 1:1:1 ratio. Reusable gel packs (20 min per session) were applied to the vastus lateralis. DOMS, maximal voluntary isometric contraction (MVIC), creatine kinase (CK), erythrocyte sedimentation rate (ESR), C-reactive protein (CRP) and ultrasound-derived muscle thickness (MT) were assessed at baseline and at 24, 48 and 72 h post-exercise and analyzed using linear mixed-effects models. Results: No condition or condition-by-time interactions were detected for the primary outcomes (DOMS, MVIC). Significant linear time effects were observed for DOMS, CK and ESR; MVIC decreased acutely and gradually recovered. A contrast-specific time-by-condition interaction for CRP did not indicate a systematic recovery advantage, and MT showed only minor temporal fluctuations. Conclusion: Neither immediate nor delayed cooling provided a meaningful recovery advantage over the control. The protocol reproducibly elicited fatigue and recovery dynamics, supporting its utility as a framework for future cryotherapy research.

## 1. Introduction

Recovery is a multifaceted restorative process (physiological and psychological) that develops over time [1]. Following high-intensity, unaccustomed or eccentric exercise, sarcomeres can undergo microstructural disruption, triggering an inflammatory cascade characterized by increased membrane permeability, infiltration of neutrophils and macrophages, and elevated circulating markers such as creatine kinase (CK) and C-reactive protein (CRP) [2,3]. These responses contribute to delayed-onset muscle soreness (DOMS), transient strength loss, and reduced functional performance typically observed 24–72 h post-exercise [4].

Localized cold therapy, such as the application of cold packs, is a widely used intervention aimed at mitigating these effects. The physiological mechanism is primarily based on vasoconstriction and decreased local blood flow, leading to reduced inflammation reactions through a decrease in cell metabolism [5]. Post-cooling-related vasoconstriction and lowered muscle tissue temperature can reduce cellular, lymphatic, and capillary permeability, thereby limiting fluid movement into the interstitial space and diminishing the likelihood of muscle fiber edema [6]. The cooling effect also decreases the conduction velocity of sensory nerves and modulates nociceptor excitability, resulting in an analgesic response that may alleviate perceived soreness [7]. Moreover, by slowing enzymatic and inflammatory processes, cold packs may reduce pro-inflammatory cytokine release and leukocyte infiltration, indirectly supporting tissue recovery.

Unlike cold-water immersion (CWI), which involves systemic exposure and hydrostatic effects, cold packs provide localized and targeted cooling without significant systemic influence. This makes them particularly relevant for clinical and rehabilitative settings, where controlled temperature application and patient comfort are critical and cold-water interventions cannot be provided. However, evidence regarding their efficacy remains mixed with respect to subjective perceived recovery and muscle soreness and objective outcomes related to strength recovery, muscle thickness, and inflammatory markers [8].

The timing of cooling may be a decisive factor in determining its physiological effects. Early application could blunt the initial inflammatory cascade, whereas delayed application may have less beneficial effects due to the build-up of a strong pro-inflammatory response [9]. Understanding the time course of cell types within skeletal muscle that contribute to muscle-immune cell interactions and regulate muscle adaptation following exercise is crucial to optimizing recovery strategies in both athletic and therapeutic contexts.

Although cold therapy is widely used to mitigate post-exercise soreness and inflammation, its optimal timing remains unclear. Immediate cooling may limit the early inflammatory response and reduce secondary muscle damage by restricting perfusion and metabolic activity during the acute post-exercise period. In contrast, delayed cooling (applied once the inflammatory response and soreness have peaked) may help alleviate discomfort and support recovery without interfering with early regenerative processes.

To address this knowledge gap, the present study directly compares the effects of immediate versus delayed local cooling on subjective and objective recovery markers following exercise-induced muscle soreness. We anticipate that immediate cooling will more strongly blunt the early pro-inflammatory response, while delayed cooling will have a greater impact on reducing muscle soreness, with both approaches outperforming a non-cooled control condition.

The primary outcomes were DOMS and maximal voluntary isometric contraction (MVIC) of the knee extensor muscles of the non-dominant leg, assessed at baseline and at 24, 48, and 72 h post-exercise. Secondary outcomes included inflammatory markers (CK, erythrocyte sedimentation rate (ESR), and CRP) and ultrasound-derived muscle thickness (MT) of the quadriceps femoris muscles (QFM), measured at the same time points. Additional monitoring included skin temperature (continuously during the first intervention using a conductive temperature logger system) and thermal perception (immediately after cold application and 20 min post-cooling, using a standardized scale ranging from −4 to +4).

Accordingly, the study addressed the following research questions. The primary research question was whether immediate or delayed local cooling, compared with a non-cooled control condition, improves recovery of DOMS and MVIC over 72 h following eccentric exercise. The secondary research questions were whether immediate or delayed cooling influences the recovery of the inflammatory markers CK, ESR, and CRP and of MT over the same period.

## 2. Materials and Methods

### 2.1. Study Design

A superiority-framed, three-arm parallel-group randomized controlled trial was conducted to investigate the effects of immediate and delayed local, repeated cold application after an exercise-induced muscle soreness protocol on subjective (DOMS) and objective (inflammation, MT, and strength recovery) characteristics during a 72 h follow-up period. The trial was conducted at the Rehabilitation and Exercise Science Laboratory (RESlab) of the University of Applied Sciences and Arts of Southern Switzerland (SUPSI) in 7302 Landquart, Switzerland; all assessments and interventions took place at this single laboratory site. The trial is reported in accordance with the CONSORT 2025 guidelines [10]; the completed CONSORT 2025 checklist is provided as Supplementary File S3.

### 2.2. Participants

A total of 48 healthy individuals from a student population (39 women, 9 men; age: 22 ± 3 years) participated in this study. Participants were allocated in a 1:1:1 ratio to one of three study groups: immediate cooling (ICG), delayed cooling (DCG; start at 24 h post-exercise), or control (CG; no intervention), according to the predefined randomization procedure. Recruitment took place between 17 March 2025 and 16 October 2025.

**Figure 1:**
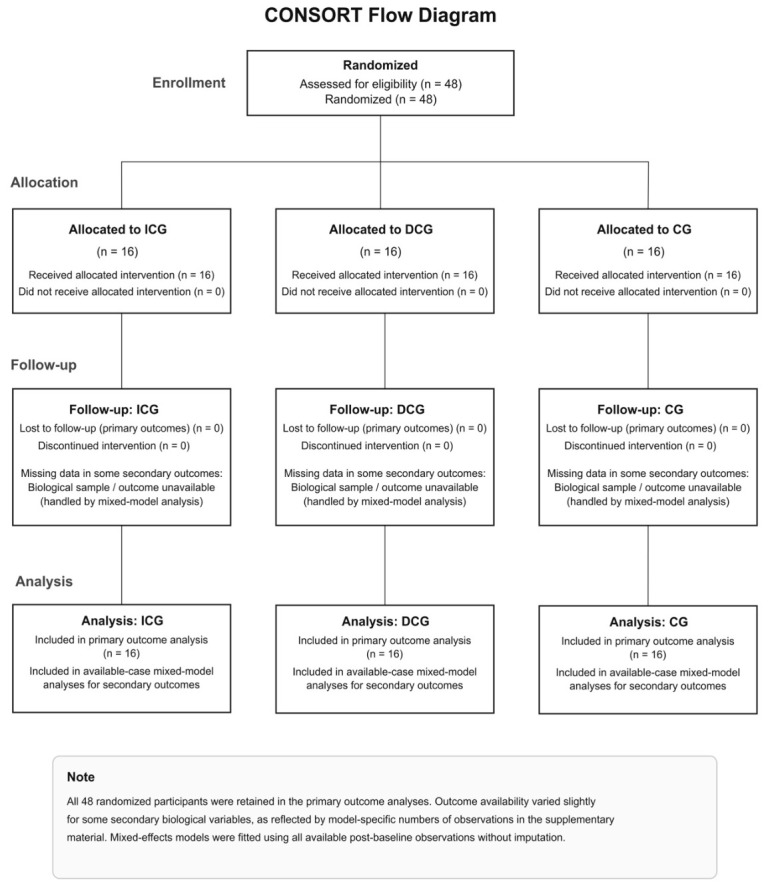
CONSORT flow diagram of participant enrollment, allocation, follow-up, and analysis.

Inclusion criteria comprised young, healthy adults aged 18–30 years with no history of surgical interventions to the musculoskeletal system. Exclusion criteria included acute pain or inflammatory conditions, medication use (excluding contraceptives), pregnancy or breastfeeding, competitive athletic activity, dermatological conditions, circulatory disorders, cold allergies (e.g., Raynaud’s syndrome), previous surgeries affecting the trunk or lower extremities, impaired sensory perception in the thigh area, diagnosed medical conditions potentially affecting participation, and smoking (all assessed via a questionnaire). All enrolled participants met eligibility criteria and were randomized. No participants were lost to follow-up. Participant flow through the study, including enrollment, allocation, follow-up, and analysis, is illustrated in Figure 1.

All participants provided written informed consent, and the study was approved by the Ethics Committee of the Canton of Zurich on 7 February 2025 (BASEC No. 2024-D0116); a substantial amendment to the protocol was subsequently approved on 11 March 2025. The trial was prospectively registered at ClinicalTrials.gov (NCT06813690; https://clinicaltrials.gov/study/NCT06813690; accessed on 20 January 2025) before the start of recruitment. This study was conducted in accordance with the Declaration of Helsinki. Patients and the public were not involved in study design, conduct, reporting or dissemination.

### 2.3. Sample Size Calculation

An a priori sample size calculation was performed using PASS 2023 (version 23.0.1; NCSS, LLC, Kaysville, UT, USA) for DOMS, one of the two primary outcomes, applying two-sided two-sample *t*-tests for the difference between each cooling group and the control group in a three-arm parallel design [11]. Planning estimates were approximated from postoperative cooling data summarized in a comprehensive review of cryotherapy [12], as directly comparable data for repeated local cooling after exercise-induced muscle soreness were limited. A control mean pain score of 4.72 and treatment means of 2.21 and 1.87 were assumed, with a common standard deviation of 2.1; these correspond to between-group differences of 2.51 and 2.85 units, equivalent to large effect sizes (Cohen’s d≈1.2 and 1.4). With a two-sided alpha of 0.05 and 80% power, the required sample size was 13 participants per group (n = 39). After allowing for an anticipated dropout rate of 10%, the recruitment target was set at 15 participants per group (total n = 45). Because the planning values were derived from postoperative rather than exercise-induced soreness data and assumed comparatively large between-group differences, the calculation should be regarded as a pragmatic planning estimate. The study was therefore powered to detect large between-group effects, while smaller effects may not have been detectable. The final analysis used linear mixed-effects models to account for repeated measurements and baseline adjustment. In practice, recruitment slightly exceeded this target, with 48 participants (16 per group) enrolled owing to the immediate availability of eligible volunteers during the recruitment window, as detailed in the protocol adherence subsection.

### 2.4. Randomization and Allocation Concealment

Participants were randomly assigned to one of three study groups in a 1:1:1 allocation ratio (ICG, DCG, or CG) using a restricted randomization procedure with a fixed allocation ratio. Allocation numbers corresponding to the study groups were prepared prior to recruitment and placed in identical opaque containers to prevent foreknowledge of the upcoming assignment, in line with established principles of allocation concealment [13]. An equal number of allocations per group was prepared to ensure balanced group sizes. After completion of baseline measurements, each participant drew one allocation to determine group assignment. Randomization occurred only after completion of all baseline assessments to prevent allocation-related measurement bias.

The allocation materials were prepared prior to recruitment by EH. Participant enrollment and baseline measurements were conducted before randomization occurred. Due to the exploratory nature and limited sample size of the study, neither block randomization nor stratification procedures were applied.

### 2.5. Blinding

Due to the nature of the intervention, blinding of participants was not feasible. However, outcome assessors conducting MVIC testing, ultrasound measurements, and blood sampling were not informed of group allocation. Standardized measurement protocols were used to further reduce potential assessment bias.

Despite these measures, the absence of participant blinding may represent a potential source of performance bias and should be considered when interpreting the findings.

### 2.6. Experimental Overview

Each participant underwent baseline measurements on day 1, including anthropometric data (body height using a GPM stadiometer, Zurich, Switzerland; body weight and estimated lower extremity body fat percentage using a TANITA-TBF 611 bioelectric impedance scale, Tokyo, Japan). After anthropometric measurements, participants were assigned to one of the study groups according to the predefined randomization procedure. Venous blood samples were collected from the antecubital vein to assess CK, ESR and CRP. Muscle thickness was evaluated in the non-dominant leg quadriceps femoris, and participants rated their subjective muscle soreness in the knee extensor muscles. MVIC of the non-dominant leg was also assessed.

**Figure 2:**
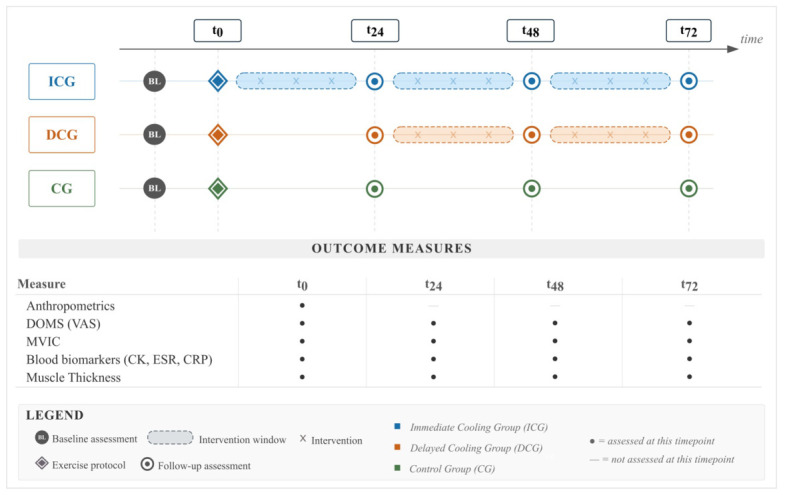
Study design and measurement schedule. Participants were randomized to immediate cooling (ICG), delayed cooling (DCG; starting 24 h post-exercise), or control (CG). Baseline assessments (BL) were conducted prior to the eccentric exercise protocol (diamond symbol). Cooling intervention windows (dashed boxes) and individual cooling sessions (X) are shown for ICG and DCG. Follow-up assessments (circles) were performed at 24, 48, and 72 h post-exercise. Outcome measures assessed at each time point are summarized in the lower panel.

Following baseline measurements, participants performed the exercise-induced muscle soreness protocol (EIMSP), comprising three warm-up sets and six work sets designed to elicit DOMS. All participants remained seated after the EIMSP; ICG participants cooled the exercised leg for 20 min, while DCG and CG participants rested without cooling. Participants returned to the laboratory at 24 h (day 2), 48 h (day 3), and 72 h (day 4) at the same time of day as baseline measurements. Environmental conditions were monitored using a digital multimeter (Voltcraft MT-52, Conrad Electronic SE, Hirschau, Germany). Figure 2 illustrates the overall experimental timeline and measurement schedule.

### 2.7. Exercise-Induced Muscle Soreness Protocol

The EIMSP was performed using a customized sitting leg-extension machine (TF-L401, Taurus/Fitshop GmbH, Schleswig, Germany). A circular plate (diameter = 20 cm) served as the rotational interface between the participant-operated movement arm and an external loading mechanism. Load was applied tangentially at the outer radius via a cable guided by a rail system, ensuring a consistent moment throughout the range of motion (ROM). Applied force was measured using an inline S-type (S-beam) load cell (5 kN rated capacity; accuracy <0.1% FS) connected to a Raspberry Pi (Raspberry Pi Ltd, Cambridge, UK), which recorded load continuously via a custom Python script (Python version 3.13; Python Software Foundation, Wilmington, DE, USA).

The protocol consisted of three warm-up sets followed by six work sets of dynamic, sitting, single-leg leg extensions, adapted from Ruas and colleagues [14]. After each set, participants rated their perceived exertion using the OMNI scale [15]. Rate of Perceived Exertion (RPE) and load from warm-up sets were used to calculate the starting weight of the first work set. Each work set targeted 12–15 repetitions at an RPE of 9–10. Load was adjusted after each set based on repetitions and RPE in steps of 5 kg. Participants performed each concentric movement explosively and controlled the eccentric phase to achieve an approximate duration of 3–4 s, continuing until they subjectively reported substantial quadriceps stretch. Participants maintained an upright seated position with their arms crossed over their chest and were secured at the waist using a seatbelt. A 1 min rest period separated all sets. Investigators provided verbal motivation and continuous verbal cues during work sets to minimize fatigue-induced form breakdown.

### 2.8. Recovery Interventions

#### 2.8.1. Cooling Procedure and Documentation

Cold therapy was applied to the vastus lateralis muscle belly using a reusable gel-based cold pack (Axanova Cold Hot Pearls Maxi Pack, 12 × 27 cm; Axanova AG, Uetliburg, Switzerland), delivered continuously for 20 min in accordance with the manufacturer’s guidelines. The cold pack was stored in a freezer for at least 24 h prior to use and positioned on a marked location with its longer edge parallel to the muscle fiber direction, secured using a bandage or towel. Participants could sit or lie down during the application.

A minimum interval of 3–4 h was required between cooling sessions, and at least 1 h elapsed between the final cooling session and each follow-up to minimize cold-induced analgesia. Participants documented each session in an online cooling diary, recording date, time elapsed since intervention, session number, and discomfort level. They also uploaded photographs verifying cold pack positioning. Women in the sample completed questionnaires regarding hormonal contraceptive use and menstrual cycle information.

Adverse events and participant discomfort were monitored non-systematically throughout the study. No formal harm definition threshold was applied; participants were instructed to cease cooling and contact the research team if they experienced pain, skin irritation, or other unexpected symptoms.

#### 2.8.2. Immediate Cooling Group

ICG participants completed a total of nine 20 min cooling sessions between baseline and 72 h post-exercise. Three sessions were completed in each 24 h interval. The first session was administered post-EIMSP immediately under supervision (t_0_). Participants then completed two additional sessions independently before the 24 h follow-up (t_24_). Following t_24_, three unsupervised sessions were conducted before the 48 h follow-up (t_48_). This pattern was repeated before the 72 h follow-up (t_72_).

#### 2.8.3. Delayed Cooling Group

DCG participants received no cooling immediately post-EIMSP. The first 20 min cooling session occurred after t_24_, followed by two independent sessions before t_48_. After t_48_, three independent sessions were completed before t_72_, totaling six sessions between 24 and 72 h post-exercise.

#### 2.8.4. Control Group

CG participants received no cooling interventions throughout the study. All follow-ups followed the same schedule as ICG and DCG.

For the duration of the 72 h follow-up, all participants were instructed to refrain from additional recovery modalities, including ice application, heat therapy, massage, anti-inflammatory medication, and strenuous physical activity. Consistent with the exclusion criteria, use of medication other than hormonal contraceptives was not permitted.

### 2.9. Outcome Assessments

#### 2.9.1. Delayed-Onset Muscle Soreness

Subjective muscle soreness was assessed using a visual analog scale (VAS) ranging from 0 cm (no soreness) to 10 cm (maximum soreness). Participants rated perceived soreness in the affected muscles while holding a squatting position with approximately 90° knee flexion [16].

#### 2.9.2. Maximal Voluntary Isometric Contraction

Participants performed three sets of maximal, isometric, seated, single-leg leg extensions at 120° knee flexion with 3 min rest between sets [17]. This assessed the MVIC of the non-dominant leg and quantified fatigue-related reductions in force production following the EIMSP. In this study, fatigue was operationally defined as the acute, transient loss of maximal force-generating capacity immediately after eccentric exercise, distinct from the prolonged strength impairments associated with structural muscle injury [18]. No motivation was provided during testing to ensure standardization.

#### 2.9.3. Venous Blood Samples

Venous blood was obtained from the antecubital fossa via venepuncture. For ESR assessment, 5.0 mL of blood was collected in VACUETTE® Erythrocyte Sedimentation Rate Tubes (2.9 mL, 3.8% sodium citrate; Greiner Bio-One, Kremsmünster, Austria) and placed upright in a VACUETTE® sedimentation stand. After resting undisturbed vertically for 1 h, ESR was measured in mm/h according to the Westergren method [19].

For CK and CRP analysis, additional samples were drawn into BD Vacutainer® SST™ II Advance serum separator tubes (8.5 mL; Becton Dickinson, Plymouth, UK). Biochemical analyses were performed by Labor Dr. Risch AG (Buchs, Switzerland). CRP concentrations were determined using a high-sensitivity immunoturbidimetric assay, while CK levels were measured using a kinetic UV method according to IFCC recommendations [20].

#### 2.9.4. Muscle Thickness

Muscle thickness of the exercised quadriceps femoris was assessed using B-mode ultrasound (MyLabClass C system, Esaote S.p.A., Genova, Italy). The minimal pressure technique measured the distance between the femoral bone and the superficial quadriceps border, excluding subcutaneous adipose tissue. Probe placement was standardized by marking the site at 60% of the distance from the greater trochanter to the lateral epicondyle, 3 cm lateral to the midline. Baseline muscle thickness values were included as covariates in the statistical models to account for inter-individual differences at study entry [21].

#### 2.9.5. Skin Temperature

Skin surface temperature of the quadriceps femoris muscle belly was continuously monitored during the EIMSP using an iButton® temperature data logger (DS1922L-F5#, Maxim Integrated, San Jose, CA, USA) at 10 Hz sampling rate. In ICG, the cold pack covered the temperature sensor during the cooling procedure to validate expected skin temperature differences following the 20 min application.

All outcomes were assessed at baseline and at 24, 48, and 72 h post-exercise using identical procedures at each time point. Follow-up evaluations were therefore performed at 24 h intervals, with all measurements recorded at the same time of day (±3 h) to minimize circadian variation. This schedule was chosen to capture the established time course of exercise-induced muscle damage, soreness, and strength loss, which typically develop over 24–72 h following eccentric exercise [3,9,18]. The same interval is appropriate for the exploratory blood markers assessed, as CK commonly rises during the first 24 h and may peak across the subsequent 24–72 h period, while CRP and ESR reflect acute-phase inflammatory kinetics with delayed responses over approximately 24–48 h [3,9,22].

### 2.10. Statistical Analysis

#### 2.10.1. Descriptive Statistics

Descriptive statistics were calculated for all anthropometric and baseline outcome variables to verify comparability across the three experimental conditions (ICG, DCG, CG). Continuous variables are reported as mean ± SD, and categorical variables as absolute and relative frequencies. Data distributions were visually screened using histograms and Q–Q plots. No formal normality hypothesis tests were performed, as linear mixed-effects models do not require normally distributed raw data, and such tests can be overly sensitive in moderate samples.

#### 2.10.2. Missing Data

Overall missingness ranged from 0–6.8% across outcomes (Table 1). No missing data occurred in the primary outcomes (DOMS, MVIC). Linear mixed-effects models provide likelihood-based estimation under a missing-at-random assumption and therefore accommodate incomplete outcome data without imputation.

#### 2.10.3. Outlier Screening

Outlier detection used ± 3 × SD z-score pooled across timepoints. Outlier prevalence was low (0.5–2.1%) and consistent with expected inter-individual variability in eccentric-exercise biomarkers (Table 2). No observations were removed.

#### 2.10.4. Group Characteristics

Anthropometric characteristics did not differ significantly between experimental conditions (Table 3). No between-group differences were observed for age, height, weight, body fat percentage, SCFL, or BMI, supporting successful randomization.

#### 2.10.5. Baseline Outcome Comparisons

No significant differences were observed at baseline for any of the primary or secondary outcomes (Table 4), confirming that groups were comparable prior to the intervention. In the primary analyses, baseline values were additionally included as covariates to adjust for any residual between-subject differences.

### 2.11. Data Processing and Quality Control

All analyses were conducted in R version 4.5.1 (R Foundation for Statistical Computing, Vienna, Austria) using a fully reproducible script and a structured workflow. The dataset was reshaped from wide to long format for repeated-measures modeling. Baseline values (t_0_) were extracted and included as outcome-specific covariates in all models (ANCOVA approach). A subject-specific random intercept was included to account for within-participant clustering of repeated observations. Given the limited number of post-baseline time points, a random-intercept structure was selected as the most parsimonious and stable specification. Percent-change values were used for visualization only and were not used as model inputs. Full fixed-effect estimates, standard errors, 95% confidence intervals, test statistics, and model diagnostics for all outcomes are reported in the Supplementary Materials.

Time was analyzed using the post-baseline measurement occasions (24, 48, and 72 h). For numerical stability and interpretability, post-baseline time was standardized (z-scored), such that the centered value corresponds to 48 h. Python was used exclusively for force acquisition during the exercise protocol.

#### Use of Generative AI Tools

During the preparation of this manuscript, the authors used generative AI assistants (ChatGPT, version GPT-5; OpenAI, San Francisco, CA, USA; and Claude, version Claude Opus 4; Anthropic, San Francisco, CA, USA) to assist with the development and debugging of the statistical analysis code in R. The conceptual analysis plan, model specifications, and interpretation of the results were defined and performed by the authors. All AI-assisted code was reviewed, tested and validated by the authors, who take full responsibility for the content of this publication.

### 2.12. Linear Mixed-Effects Modeling

Longitudinal trajectories in DOMS, MVIC, CK, ESR, CRP, and MT were analyzed using linear mixed-effects models (LMMs) with subject-specific random intercepts (1 | id). Fixed effects included condition (sum-to-zero contrasts), standardized time, and their interaction. Baseline outcome value, sex, and subcutaneous fat layer thickness (SCFL) were included as covariates.

For each outcome, linear and quadratic time specifications were compared using maximum likelihood estimation. Model comparison was based on likelihood ratio tests and the Akaike Information Criterion (AIC). A quadratic term was retained only if it improved model fit according to both criteria. In all outcomes, the linear time specification provided the more parsimonious fit and was therefore retained. Final parameter estimates and inference were obtained from models refitted using restricted maximum likelihood (REML).

CRP was analyzed on the log scale (log(CRP)) due to strong right-skew; both the post-baseline outcome and its baseline covariate were log-transformed. To improve numerical stability, models were fitted using the bobyqa optimizer. A sensitivity analysis for CRP was conducted by refitting the final model after excluding the participant with the highest baseline log(CRP) in order to evaluate the robustness of the time-by-condition interaction. Additionally, sensitivity analyses were performed for CK and ESR using log-transformed outcomes (log1p) and corresponding baseline covariates to evaluate robustness against right-skewed distributions. These analyses did not materially alter the substantive conclusions: no condition or condition-by-time effects were detected in either parameterization. For CK, the linear time effect remained highly significant on the log1p scale (p<0.001); for ESR, the linear time effect weakened from p=0.030 on the raw scale to p=0.099 on the log1p scale, consistent with modest skew-induced inflation in the raw analysis, while the absence of condition and condition-by-time effects was preserved.

All randomized participants were included in the primary analysis according to the intention-to-treat principle, with all available observations retained in the linear mixed-effects models. Minor missingness occurred only in some secondary biological outcomes.

### 2.13. Handling of Multiple Comparisons

The primary outcomes were DOMS and MVIC. For each primary outcome, the primary between-group inference was based on the omnibus condition-by-time interaction from the mixed-effects model. No multiplicity correction was applied to these primary model-based analyses. As a secondary descriptive step for the primary outcomes, pairwise condition contrasts were computed from estimated marginal means evaluated at the centered post-baseline timepoint (time_std = 0; corresponding to 48 h), and Holm correction was applied across the three pairwise contrasts within each primary outcome.

Secondary outcomes (CK, ESR, CRP, MT) were analyzed in an exploratory manner and are reported without multiplicity correction. Given the exploratory character of these secondary outcomes and the number of tested endpoints, these results should be interpreted cautiously.

### 2.14. Model Diagnostics

Model assumptions were evaluated using simulation-based residual diagnostics (DHARMa), including assessments of residual dispersion, uniformity, and outliers. Variance inflation factors (VIF) were examined to screen for multicollinearity among fixed effects. All final models achieved numerical convergence, and no singular random-effects structures were observed. Dispersion tests indicated no over- or under-dispersion for any outcome (all *p* > 0.6). Mild departures from residual uniformity were detected for some outcomes (DOMS, CK, CRP, ESR; uniformity-test *p*< 0.05), consistent with the right-skewed distributions characteristic of inflammatory and tissue-stress markers. CRP was therefore analyzed on the natural log scale, and sensitivity analyses for CK and ESR were performed using log1p-transformed values; these did not materially alter the substantive conclusions, supporting the robustness of the primary inference.

### 2.15. Protocol Adherence

The study was conducted in accordance with the prospectively approved Clinical Investigation Plan (Version 06, 3 March 2025) and the trial registration (NCT06813690). Recruitment slightly exceeded the planned sample size (48 vs. planned 45 participants) due to the availability of eligible volunteers. Minor missing data occurred for some secondary biological outcomes due to occasionally unavailable samples; however, all available observations were retained in the linear mixed-effects analyses without imputation. The final statistical analysis used linear mixed-effects models to account for repeated measurements and baseline adjustment, consistent with the planned analytical framework while improving statistical efficiency. No deviations affecting eligibility criteria, primary outcomes, or study conclusions occurred.

## 3. Results

### 3.1. Participant Flow

A total of 48 participants were enrolled and randomized equally to the immediate cooling group (ICG, n = 16), delayed cooling group (DCG, n = 16), and control group (CG, n = 16). The sample was predominantly composed of women (39 women, 9 men; 81% women), reflecting the demographic composition of the student cohort from which participants were recruited. All participants completed follow-up assessments for the primary outcomes, and no participants were lost to follow-up or discontinued the intervention. No adverse events occurred during the study.

Minor missing data occurred for some secondary biological outcomes due to occasionally unavailable samples; however, all available observations were retained in the linear mixed-effects analyses without imputation.

### 3.2. Overview

The primary aim of this study was to determine whether recovery trajectories differed between ICG and DCG following the EIMSP over a 72 h period. Sensitivity analyses for CRP yielded comparable effect directions and magnitudes, supporting the robustness of the CRP results.

**Figure 3:**
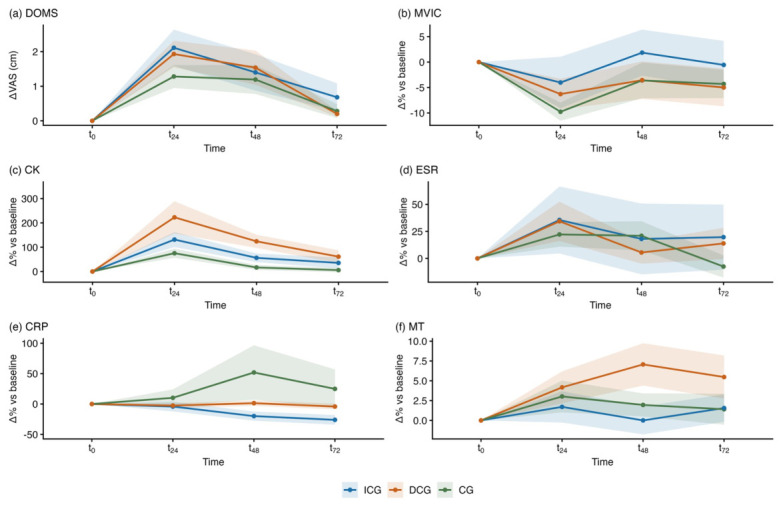
Change from baseline in recovery markers over 72 h following eccentric exercise by condition. Mean change from baseline (t_0_). Panel (**a**) shows absolute change in DOMS (ΔVAS, cm); panels (**b**–**f**) show percent change relative to baseline for MVIC, CK, ESR, CRP, and MT. Lines represent group means for immediate cooling (ICG), delayed cooling (DCG), and control (CG); shaded areas indicate ±1 SD. Time points: t_0_, t_24_, t_48_, and t_72_.

Recovery patterns over time were evident for several outcomes, but no consistent between-condition differences were found for the primary outcomes. Significant linear time effects were observed for DOMS, CK, ESR, and CRP, indicating systematic post-exercise changes across the 72 h follow-up period. MVIC and MT did not demonstrate statistically significant linear time effects. Neither immediate nor delayed cooling altered these trajectories relative to the control condition (Figure 3).

For the primary outcome, DOMS, a significant linear time effect was detected, reflecting increasing soreness followed by recovery across the 72 h period. MVIC showed a visible dip at 24 h followed by recovery; however, the linear time effect did not reach statistical significance. Among the secondary outcomes, CK, ESR, and CRP exhibited statistically significant linear time effects, although the effect magnitudes were small, and no consistent between-condition recovery advantage was observed. Sensitivity analysis for CRP, excluding the participant with the highest baseline log(CRP), did not materially alter the time-by-condition inference.

In summary, neither immediate nor delayed cooling provided a significant recovery advantage over the control condition for any measured variable.

### 3.3. Primary Outcomes

No significant condition or condition-by-time interaction effects were found for either primary outcome (Table 5). DOMS showed a significant linear time effect across the 72 h follow-up period, whereas MVIC did not demonstrate a statistically significant linear time effect. These results indicate changes in soreness over time following the exercise protocol, without evidence of differential recovery between the cooling conditions. Alternatively, the magnitude of the cooling intervention may have been insufficient to produce detectable effects, particularly for objectively measured physiological outcomes that may require larger or repeated stimuli to show measurable changes.

#### 3.3.1. DOMS

A significant linear time effect was observed for DOMS (β = −0.568±0.080, *p*<0.001), indicating systematic change in soreness across the 24–72 h follow-up period. No significant condition or time-by-condition interaction effects were detected, suggesting comparable recovery trajectories in ICG, DCG and CG (Figure 3a).

#### 3.3.2. MVIC

MVIC did not demonstrate a statistically significant linear time effect (β = 2.000±1.365, *p* = 0.146). Recovery trajectories did not differ between ICG, DCG, and CG.

The model intercept (β = 74.152 ± 16.828, *p*< 0.001) represents the expected MVIC value at the centered post-baseline time point (48 h), conditional on covariates. Baseline MVIC was a strong predictor of follow-up strength values (β = 0.628 ± 0.075, *p*< 0.001).

### 3.4. Secondary Outcomes

#### 3.4.1. CK

CK demonstrated a statistically significant linear time effect (β = −56.940±7.582, *p* < 0.001), indicating temporal change across the 24–72 h follow-up period. No significant overall condition effect or time-by-condition interaction was detected.

#### 3.4.2. ESR

ESR demonstrated a modest but statistically significant linear time effect (β = −0.450±0.205, *p* = 0.030). No significant condition or time-by-condition interaction effects were observed.

#### 3.4.3. CRP

CRP, analyzed on the natural log scale, demonstrated a significant linear time effect (β = −0.057±0.015, *p*< 0.001) and an omnibus time-by-condition interaction (Type III Wald $\chi^{2}$, *p* = 0.004). Under sum-to-zero contrasts, the significant interaction signal was concentrated in the ICG contrast (ICG vs. grand mean: β = −0.073±0.022, *p* = 0.001); the CG contrast was not significant (β = 0.026±0.021, *p* = 0.222), and the DCG contrast (implied by the sum-to-zero constraint) was β = 0.046. No significant overall main effect of condition was detected. The interaction signal was therefore localized to a single contrast rather than reflecting a coherent between-condition pattern and did not indicate a systematic recovery advantage. Given the exploratory nature of secondary outcomes, these findings were interpreted cautiously. Sensitivity analysis excluding the participant with the highest baseline log(CRP) did not materially alter the pattern of results.

#### 3.4.4. MT

MT did not change significantly over the 72 h recovery period. No main effects of time or condition and no interaction effects were detected, indicating stable muscle thickness across groups (Figure 3f).

Overall, exploratory analyses of secondary outcomes did not reveal consistent or robust between-condition differences. Temporal variations observed in certain biomarkers reflected expected post-exercise responses rather than cooling-related recovery advantages.

### 3.5. Summary of Findings

Across all documented cooling sessions, discomfort was reported infrequently (overall rate 6.6%). The highest proportion occurred at 24 h post-intervention (9.2%), with lower rates at 48 h (4.1%) and 72 h (7.0%). Reported discomfort was similar across sessions involving one, two, or three cooling applications prior to follow-up (range: 6.3–7.7%), and no clear descriptive pattern was evident across timepoints or number of prior cooling sessions.

## 4. Discussion

The present study investigated whether immediate or delayed local cold exposure influences recovery following exercise-induced muscle fatigue. No significant condition or condition-by-time effects were detected for the primary outcomes (DOMS, MVIC). Secondary outcomes (CK, ESR, CRP) showed exploratory time-dependent changes, and CRP demonstrated a contrast-specific interaction signal; however, these effects were small, not consistent across all contrasts, and did not indicate a clear or systematic benefit of immediate or delayed cooling.

Clear time-dependent changes confirmed that the eccentric leg-extension protocol effectively induced muscular fatigue and subsequent recovery. DOMS increased markedly within 24–48 h, while MVIC decreased at 24 h post-exercise and progressively recovered. Similarly, CK, ESR, and CRP exhibited statistically significant linear time effects consistent with expected post-exercise physiological responses. Ultrasound-measured muscle thickness did not demonstrate a significant temporal change. These findings indicate that the experimental model elicited measurable fatigue-recovery dynamics over time.

Current research presents mixed findings regarding the effectiveness of cold therapy in enhancing performance recovery [23]. Consistent with the present findings, Jakeman and colleagues reported that a single cold-water immersion following intense plyometric exercise did not significantly restore concentric muscle strength [24]. One possible explanation is that a single cold exposure may be insufficient to meaningfully influence the physiological processes underlying exercise-induced muscle damage. Importantly, even repeated applications of cryotherapy following muscle-damaging exercise have frequently failed to demonstrate clear functional recovery benefits [25,26]. This aligns with the present data, in which neither immediate nor delayed cooling altered strength recovery or soreness trajectories despite observable time-dependent biomarker changes. It is also conceivable that high-intensity eccentric or plyometric exercise induces structural and inflammatory responses of sufficient magnitude that external cooling interventions, despite their high thermal conductivity, exert only limited influence on the downstream mechanisms governing performance recovery [27].

Therefore, the current study protocol was designed to induce pronounced exercise-induced muscle soreness without provoking extensive structural muscle damage. The eccentric-based protocol by Ruas and colleagues was used as the conceptual foundation to elicit sufficient soreness [14]. In their study, the concentric-eccentric exercise group demonstrated a substantial MVIC reduction of 23.6 ± 23.3% at days 1-3 compared to baseline (*p*≤ 0.001). In contrast, the present study showed only a modest descriptive reduction in MVIC within the control group across 24-72 h (mean change ≈ 6%; Figure 3b), and the mixed-effects analysis did not detect a statistically significant linear time effect. This suggests that, although the protocol reliably induced subjective soreness and biomarker responses, the magnitude of functional impairment was comparatively moderate. Previous literature indicates that unaccustomed eccentric-only contractions typically produce greater structural muscle disruption and prolonged force deficits, whereas concentric-dominant loading induces comparatively minor impairments [28,29]. The present findings therefore suggest that the protocol elicited measurable fatigue and soreness responses without evidence of severe or prolonged strength loss.

The exercise protocol was designed to induce a pronounced soreness response, conceptually based on the model proposed by Ruas and colleagues [14]. In the present study, participants performed six work sets of 12–15 repetitions combining concentric and eccentric contractions, whereas Ruas and colleagues employed eight alternating concentric and eccentric contractions. Although the total number of repetitions in our protocol was higher, differences in load prescription may have influenced the overall mechanical stimulus. Specifically, Ruas and colleagues adjusted inter-set loads in smaller increments (1–3 kg), allowing more precise targeting of the intended relative intensity. In contrast, load adjustments in the present study were limited to 5 kg intervals due to the mechanical configuration of the ergometer chair. Furthermore, Ruas and colleagues were able to prescribe both concentric and eccentric workloads at 80% of MVIC, whereas in the current setup, only concentric 80% MVIC could be quantified directly. As a result, eccentric loading intensity may have varied more between participants. These methodological differences may partly explain the comparatively modest MVIC reductions observed in the present study despite the higher repetition volume.

The absence of significant condition effects may partly reflect the localized and superficial nature of the cooling intervention. Reusable gel packs primarily reduce skin and subcutaneous tissue temperature, and their capacity to meaningfully alter intramuscular temperature depends strongly on adipose tissue thickness. Although cooling was standardized across participants, inter-individual differences in subcutaneous fat layer thickness (SCFL) may have attenuated heat transfer to deeper muscle tissue. Subcutaneous adipose tissue is a well-established determinant of intramuscular temperature change during cryotherapy [30,31]. Myrer and colleagues demonstrated that application of a crushed-ice pack to the calf for 20 min reduced intramuscular temperature at 1 cm depth by 14.43 °C, 9.06 °C, and 5.00 °C in individuals with skinfold thicknesses of ≤8 mm, 10–18 mm, and ≥20 mm, respectively. At 3 cm depth, the corresponding temperature reductions were substantially smaller (6.22 °C, 3.86 °C, and 2.42 °C) [31]. These findings suggest that even moderate differences in adiposity can markedly influence intramuscular cooling magnitude. It should be noted, however, that these observations were obtained under resting conditions and may not directly translate to post-exercise muscle tissue, where increased blood flow and metabolic activity can accelerate rewarming. Furthermore, the analgesic effects of cryotherapy are closely linked to reductions in tissue temperature sufficient to decrease sensory nerve conduction velocity [32]. Experimental evidence suggests that clinically meaningful analgesia typically requires tissue temperature reductions in the range of approximately 5–15 °C, with skin temperature often needing to fall below roughly 12 °C [33]. Whether the present intervention achieved such intramuscular temperature thresholds consistently across participants remains uncertain.

These findings align with previous research indicating that local cryotherapy or cold packs exert limited effects on objective recovery markers when compared with passive rest or whole-body immersion [6,34,35]. Whereas systemic water cooling can reduce core and intramuscular temperatures and modulate inflammation and neuromuscular function [36,37], local cooling effects are typically smaller due to reduced surface area contact, lower thermal conductivity, and the absence of hydrostatic pressure [38,39,40,41]. Consequently, local cooling may alleviate perceived discomfort without producing measurable changes in objective markers of tissue repair or functional recovery. In the present study, CK, CRP, and ESR demonstrated significant time-dependent changes, reflecting expected post-exercise physiological responses. However, these trajectories were not consistently modified by either immediate or delayed cooling. The isolated time-by-condition signal observed for CRP was not accompanied by coherent effects across related inflammatory or structural markers and therefore does not support a robust physiological cooling effect. Overall, inflammatory and remodeling processes appeared to progress without meaningful modulation by the localized cooling intervention.

From a mechanistic standpoint, the recovery patterns observed across markers reflect normal physiological adaptation to eccentric loading rather than treatment-dependent modulation. Significant time-dependent changes in DOMS, CK, ESR, and CRP indicate activation and resolution of inflammatory and repair-related processes following muscle loading. The concurrent restoration of MVIC and reduction of DOMS likely represent neural and mechanical recalibration as muscle function normalizes. Importantly, these trajectories were not consistently altered by either immediate or delayed cooling. The limited influence of cold exposure observed in this study suggests that superficial temperature reduction may not meaningfully affect deeper cellular processes involved in muscle repair, such as protein synthesis, satellite-cell activation, or perfusion-mediated waste clearance [9,42].

Cooling may also influence neuromuscular function through mechanisms not captured by the present outcomes. Reductions in muscle and nerve temperature slow nerve conduction velocity and may alter motor unit recruitment and firing rates and shift the electromyographic (EMG) frequency spectrum, which can transiently modify force production and muscle activation [32,41]. Following exercise-induced muscle damage, however, studies that have directly quantified these processes report limited benefit: Guilhem and colleagues applied repeated cryotherapy after eccentric exercise and found no group differences in EMG activity, creatine kinase, or perceived soreness, although cooling transiently delayed changes in muscle activation and oedema [43].

The present strength findings are consistent with this pattern, but because neuromuscular activation was not recorded, the extent to which motor unit recruitment or neural drive contributed to the observed MVIC trajectories cannot be determined.

From an applied perspective, the findings indicate that localized cooling with gel packs is safe, feasible, and well tolerated after intensive lower-limb exercise, but no evidence was found for accelerated physiological or functional recovery. In rehabilitation or physiotherapy contexts, such localized cooling may still serve as a useful adjunct for pain relief or perceived recovery when systemic cooling is impractical or contraindicated. For athletes or clinical populations aiming to influence deeper inflammatory or repair-related processes, more comprehensive modalities such as cold-water immersion, controlled cryotherapy systems, or heat-based interventions may be more appropriate, as suggested by previous work [8].

In summary, localized cold application may enhance comfort, particularly in relation to perceived muscle soreness, but appears to exert limited influence on objective markers of physiological recovery following eccentric exercise. These findings underscore the importance of tailoring cooling strategies to specific recovery goals (such as symptom management in physiotherapy, localized edema control in rehabilitation, or systemic recovery enhancement in athletic contexts), ensuring that the selected modality aligns with the intended depth and mechanism of action.

### 4.1. Study Limitations

Several limitations should be considered when interpreting these findings. First, the majority of participants were women, which may have influenced both thermal sensitivity and mechanical responses [44]. Greater subcutaneous fat thickness in the thigh region in women compared with men, particularly above the vastus lateralis, could have attenuated penetration of the cooling stimulus into deeper muscle tissue [45]. Although SCFL was included as a covariate in the statistical models, the relatively homogeneous sample of healthy young adults limits generalizability to trained athletes, older adults, or clinical populations.

Second, the study’s sample size (n = 48, 16 per group) and the observed between-subject variability (ICC values ranged from approximately 0.47 to 0.81 across outcomes) may have reduced sensitivity to detect small-to-moderate between-group differences. While the design was likely sufficient to detect large treatment effects, smaller effects, particularly for exploratory secondary outcomes, may have gone undetected. Accordingly, non-significant findings should not be interpreted as definitive evidence of the absence of effect.

Third, the cooling modality consisted of reusable gel packs, which provide localized and surface-limited cooling compared with more intensive approaches such as cold-water immersion or controlled cryotherapy systems. The thermal dose delivered to deeper muscle tissue may therefore have been insufficient to meaningfully modulate inflammatory or regenerative processes.

Additionally, although the application procedure was standardized and participants received detailed instructions, the predominantly self-administered cooling sessions may have introduced inter-individual variability in cold pack positioning, compression, and protocol adherence. This potential variability in intervention delivery may have reduced treatment fidelity and contributed to increased response variability.

Finally, no direct measures of intramuscular temperature, continuous skin temperature during recovery, or local perfusion were obtained following the intervention. Although skin temperature was monitored during the exercise protocol, the depth and duration of post-intervention tissue cooling cannot be fully verified. Future research should employ larger and more diverse samples, standardized cooling modalities with quantifiable thermal dosing, and integrated temperature or perfusion monitoring to more precisely characterize the physiological impact of localized cold exposure.

In addition, recovery was characterized using subjective soreness, maximal voluntary strength, circulating biomarkers, and muscle thickness, but no EMG or other neuromuscular assessments were obtained. Consequently, the present data cannot establish whether cooling influenced muscle activation patterns, motor unit recruitment, or neural recovery strategies. Although MVIC was expressed as a percent change from each participant’s baseline, and baseline MVIC was included as a covariate in the statistical models, these procedures only adjust for between-subject differences in strength and do not substitute for direct neuromuscular measurement. Therefore, the absence of group differences in MVIC should not be interpreted as evidence that neuromuscular activation was unaffected [41,43].

### 4.2. Future Directions

Future studies should expand upon these findings by addressing the identified limitations and refining methodological precision. Increasing sample size and achieving a more balanced sex distribution would enhance statistical sensitivity and external validity, enabling exploration of potential sex-specific responses to cold exposure. Including trained athletes, older adults, or clinical populations may further clarify whether baseline fitness, muscle composition, or recovery capacity modulates responsiveness to localized cooling interventions.

From an intervention standpoint, future research should incorporate deeper or more uniformly distributed cooling modalities (such as cold-water immersion or controlled cryotherapy systems) to achieve more consistent tissue temperature reductions across muscle layers. The integration of direct physiological monitoring, including intramuscular thermocouples, near-infrared spectroscopy, laser Doppler flowmetry, or infrared thermography, would allow verification of cooling depth and associated changes in perfusion and metabolic activity. Quantifying the delivered thermal dose would improve mechanistic interpretation and intervention fidelity.

Given the substantial between-subject variability observed across outcomes, future protocols may benefit from individualized cooling strategies. Tailoring exposure parameters (e.g., duration or intensity) to achieve predefined physiological targets (such as a standardized percentage reduction in skin or intramuscular temperature) could reduce heterogeneity in thermal response and improve comparability across participants.

Finally, future studies should incorporate surface EMG or other neuromuscular assessments alongside strength and soreness measures to clarify whether cooling influences muscle activation, motor unit recruitment, or neural aspects of recovery that may not be reflected in force-based outcomes alone. Extending the observation window beyond 72 h and adding further performance-based markers (such as jump performance, rate-of-force development, or functional strength assessments) would additionally capture later phases of recovery and provide a more comprehensive understanding of how localized cold exposure influences both short-term recovery dynamics and longer-term adaptation following eccentric loading.

### 4.3. Practical Implications

From a practical perspective, the present findings indicate that local cold application using reusable gel packs can be safely implemented following intensive lower-limb exercise and may enhance comfort or perceived recovery; however, no evidence was found for accelerated physiological recovery under the tested conditions. Given that the exercise protocol induced measurable but controlled fatigue without pronounced muscle damage, localized cooling may be most relevant in contexts where short-term soreness modulation or comfort management is prioritized over deep tissue temperature reduction.

Practitioners should recognize that localized surface cooling produces limited intramuscular temperature changes, particularly in individuals with greater subcutaneous fat thickness. When the objective is to meaningfully influence systemic or deeper physiological recovery processes, more comprehensive modalities (such as cold-water immersion or other immersion-based cooling strategies) may be required. In applied rehabilitation or athletic settings, local cryotherapy may therefore be best positioned as a complementary recovery modality aimed at comfort and perceived readiness rather than as a primary intervention for accelerating structural or functional muscle restoration. The practical interpretation of these findings is limited to the variables measured (soreness, strength, inflammatory markers, and muscle thickness) and should not be extended to neuromuscular adaptations, such as changes in muscle activation or motor unit behavior, which were not assessed in the present study.

## 5. Conclusions

Immediate and delayed local cold exposure did not significantly modify recovery trajectories following eccentric exercise, as no condition or condition-by-time effects were detected for the primary outcomes. The exercise protocol successfully induced measurable fatigue and significant time-dependent changes in several recovery markers, confirming its suitability as a reproducible experimental framework.

Although localized cooling was well tolerated and may enhance comfort or perceived recovery, no evidence was found for accelerated physiological recovery under the tested conditions. For the outcomes assessed (soreness, strength, inflammatory markers, and muscle thickness), superficial cooling did not meaningfully influence recovery after eccentric loading. These findings do not, however, extend to neuromuscular or neural recovery processes, which were not measured. The experimental model provides a reproducible platform for future investigations into recovery strategies and the physiological mechanisms underlying exercise-induced muscle adaptation.

## Figures and Tables

**Table 1:** Missing data summary across outcomes.

Variable	Missing (n)	Percent Missing
ESR	13	6.77%
CK	6	3.12%
CRP	6	3.12%
DOMS	0	0.00%
MT	0	0.00%
MVIC	0	0.00%

Values reflect total missing observations across all timepoints (t_0_, t_24_, t_48_, t_72_; n = 48 participants, 192 possible observations per outcome). Linear mixed-effects models were fitted under the Missing At Random (MAR) assumption.

**Table 2:** Outlier summary across outcomes.

Variable	Total Obs.	Outliers (n)	Percent
ESR	179	4	2.23%
CK	186	3	1.61%
CRP	186	2	1.08%
DOMS	192	2	1.04%
MT	192	1	0.52%
MVIC	192	0	0.00%

Outlier detection used ±3 SD z-score pooled across timepoints. No observations were removed or winsorized; values are reported for descriptive quality control only. Total observations reflect non-missing values per outcome.

**Table 3:** Anthropometric and baseline characteristics by group.

Group	n	Age	Height (m)	Weight (kg)	BFP (%)	SCFL (mm)	BMI
ICG	16	22.81 ± 3.15	1.66 ± 0.08	61.33 ± 13.53	28.06 ± 5.27	8.7 ± 2.7	22.18 ± 3.68
DCG	16	22.69 ± 2.44	1.67 ± 0.08	62.52 ± 9.91	26.88 ± 6.11	7.0 ± 3.8	22.22 ± 2.31
CG	16	22.81 ± 3.23	1.70 ± 0.08	69.26 ± 13.20	30.75 ± 6.88	8.6 ± 3.4	23.78 ± 3.91
*p*-value	–	0.989	0.287	0.196	0.255	0.308	0.377

Values are presented as mean ± SD. *p*-values from Welch one-way tests. SCFL = mean thigh skinfold thickness measured by caliper and expressed in millimeters. BFP = body fat percentage.

**Table 4:** Baseline (t_0_) outcome comparison across conditions.

Group	${\mathrm{DOMS}}_{t_{0}}$	${\mathrm{MVIC}}_{t_{0}}$	${\mathrm{Ck}}_{t_{0}}$	${\mathrm{ESR}}_{t_{0}}$	${\mathrm{CRP}}_{t_{0}}$	${\mathrm{MT}}_{t_{0}}$
ICG	0.11 ± 0.35	169.57 ± 49.47	180.47 ± 172.16	8.00 ± 8.29	3.97 ± 11.05	3.85 ± 0.76
DCG	0.10 ± 0.17	183.17 ± 46.89	116.31 ± 72.00	4.62 ± 2.87	0.67 ± 0.36	3.57 ± 0.64
CG	0.25 ± 0.55	192.91 ± 32.86	140.06 ± 56.86	5.93 ± 2.81	1.74 ± 1.76	3.99 ± 0.61
*p*-value	0.590	0.309	0.350	0.231	0.053	0.185

Values are presented as mean ± SD. *p*-values from Welch one-way tests.

**Table 5:** Summary of linear mixed-effects model results for all outcome variables.

Outcome	Time Effect	Condition Effect	Interaction	ICC
DOMS (linear)	*p* < 0.001 **	ns	ns	0.617
MVIC (linear)	ns	ns	ns	0.468
CK (linear)	*p* < 0.001 **	ns	ns	0.593
ESR (linear)	*p* = 0.028 *	ns	ns	0.476
CRP (linear)	*p* < 0.001 **	ns	*p* = 0.004 **	0.809
MT (linear)	ns	ns	ns	0.522

Displayed are omnibus Type III Wald $\chi^{2}$ tests for the main effects of time, condition, and the time-by-condition interaction based on the final selected linear time specification. ICC values are derived from the random-intercept models. Holm correction was applied only to post hoc pairwise contrasts for the primary outcomes and is not reflected in the omnibus tests shown here. Secondary outcomes are reported as exploratory without multiplicity correction. ** *p*< 0.01, * *p*< 0.05, ns = not significant.

## Data Availability

The original contributions presented in this study are included in the article and its Supplementary Materials. The Clinical Investigation Plan (Version 06, 3 March 2025) and the full statistical analysis code are provided as Supplementary Materials with this article. Further inquiries can be directed to the corresponding author.

## Supplementary Materials

The following supporting information can be downloaded at: https://www.mdpi.com/article/10.3390/ijerph23070887/s1, File S1: Statistical analysis code. Fully reproducible R script used for data preprocessing, model specification and selection, linear mixed-effects modeling, multiplicity adjustment for primary outcomes, sensitivity analyses, and model diagnostics. File S2: Clinical Investigation Plan. Full study protocol (Version 06, 3 March 2025) including eligibility criteria, randomization procedures, intervention protocols, outcome assessment procedures, and the pre-specified statistical analysis framework. File S3: CONSORT 2025 checklist. Completed CONSORT 2025 reporting checklist for randomized controlled trials. Table S1: Full linear mixed-effects model output. Complete fixed-effect estimates, standard errors, 95% confidence intervals, test statistics, raw *p*-values, intraclass correlation coefficients (ICC), model fit characteristics, and convergence diagnostics for all outcomes. Fixed-effect estimates are derived from the final REML models with Satterthwaite degrees of freedom. Multiplicity correction was applied only to post hoc pairwise contrasts for primary outcomes and is not reflected in this table. Data S1: Raw dataset. Participant-level data for all outcomes across timepoints used in the linear mixed-effects analyses.

## Abbreviations

The following abbreviations are used in this manuscript:

BFP	Body fat percentage
BMI	Body mass index
CG	Control group
CK	Creatine kinase
CRP	C-reactive protein
CWI	Cold-water immersion
DCG	Delayed cooling group
DOMS	Delayed-onset muscle soreness
EIMSP	Exercise-induced muscle soreness protocol
ESR	Erythrocyte sedimentation rate
ICG	Immediate cooling group
ICC	Intraclass correlation coefficient
MAR	Missing at random
MT	Muscle thickness
MVIC	Maximal voluntary isometric contraction
QFM	Quadriceps femoris muscle
ROM	Range of motion
RPE	Rate of perceived exertion
SCFL	Subcutaneous fat layer thickness
