# Supplementary material for: Effects of Immediate and Delayed Repeated Cold Exposure After Physical Exertion: A Randomized Controlled Trial
Source: Int J Environ Res Public Health. 2026 Jul 9;23(7):887. doi: 10.3390/ijerph23070887 (PMC13410065; doi:10.3390/ijerph23070887)
Supplement: Supplementary file 1 [file ijerph-23-00887-s001.zip › TableS1_Full linear mixed-effects model output.pdf]

Table S1. Full linear mixed-effects model output and diag-nostics (with 95% CIs). Complete fixed-effect estimates, standard errors, 95% confidence intervals, test statistics, *p*-values, and model diagnostics for all outcomes. All models used a linear time specification with sum-to-zero factor contrasts.

Outcome	Fixed effect term	Est.	SE	95% CI	t	df	p	Note	ICC	Sing.	Conv.	n_{subj}	n_{obs}
DOMS	(Intercept)	1.614	0.555	[0.516, 2.711]	2.908	42.0	0.006	Grand mean (intercept)	0.617	False	True	48	144
DOMS	time_std	-0.568	0.080	[-0.728, -0.409]	-7.065	93.0	2.89×10^{-10}	Primary outcome	0.617	False	True	48	144
DOMS	condition1	-0.206	0.281	[-0.761, 0.349]	-0.734	42.0	0.467	Primary outcome	0.617	False	True	48	144
DOMS	condition2	0.185	0.277	[-0.364, 0.733]	0.666	42.0	0.509	Primary outcome	0.617	False	True	48	144
DOMS	DOMS_baseline	0.369	0.546	[-0.712, 1.449]	0.674	42.0	0.504	Primary outcome	0.617	False	True	48	144
DOMS	sex1	0.681	0.264	[0.159, 1.202]	2.582	42.0	0.013	Primary outcome	0.617	False	True	48	144
DOMS	SCFL	0.011	0.061	[-0.110, 0.132]	0.181	42.0	0.857	Primary outcome	0.617	False	True	48	144
DOMS	time_std:condition1	0.159	0.114	[-0.066, 0.384]	1.395	93.0	0.166	Primary outcome	0.617	False	True	48	144
DOMS	time_std:condition2	-0.018	0.114	[-0.243, 0.207]	-0.158	93.0	0.875	Primary outcome	0.617	False	True	48	144
MVIC	(Intercept)	74.152	16.828	[40.867, 107.436]	4.407	42.0	7.12×10^{-5}	Grand mean (intercept)	0.468	False	True	48	144
MVIC	time_std	2.000	1.365	[-0.700, 4.700]	1.465	93.0	0.146	Primary outcome	0.468	False	True	48	144
MVIC	condition1	1.795	3.783	[-5.688, 9.277]	0.474	42.0	0.638	Primary outcome	0.468	False	True	48	144
MVIC	condition2	0.790	3.818	[-6.762, 8.342]	0.207	42.0	0.837	Primary outcome	0.468	False	True	48	144
MVIC	MVIC_baseline	0.628	0.075	[0.479, 0.777]	8.329	42.0	1.95×10^{-10}	Primary outcome	0.468	False	True	48	144
MVIC	sex1	5.396	4.121	[-2.754, 13.547]	1.310	42.0	0.197	Primary outcome	0.468	False	True	48	144
MVIC	SCFL	-1.597	0.814	[-3.206, 0.013]	-1.962	42.0	0.056	Primary outcome	0.468	False	True	48	144
MVIC	time_std:condition1	2.110	1.930	[-1.708, 5.928]	1.093	93.0	0.277	Primary outcome	0.468	False	True	48	144
MVIC	time_std:condition2	-0.361	1.930	[-4.179, 3.457]	-0.187	93.0	0.852	Primary outcome	0.468	False	True	48	144

Continued on next page

Table 1: **S1 Table.** Full linear mixed-effects model output and diagnostics (continued).

**Outcome**	**Fixed effect term**	**Est.**	**SE**	**95% CI**	t	**df**	p	**Note**	**ICC**	**Sing.**	**Conv.**	n_{subj}	n_{obs}
CK	(Intercept)	99.150	51.242	[-2.241, 200.540]	1.935	41.4	0.059	Grand mean (intercept)	0.593	False	True	47	139
CK	time_std	-56.940	7.582	[-71.943, -41.937]	-7.510	89.5	4.24×10^{-11}	Exploratory (secondary)	0.593	False	True	47	139
CK	condition1	-49.976	24.608	[-98.667, -1.285]	-2.031	41.1	0.049	Exploratory (secondary)	0.593	False	True	47	139
CK	condition2	2.846	25.742	[-48.089, 53.781]	0.111	41.8	0.913	Exploratory (secondary)	0.593	False	True	47	139
CK	CK_baseline	1.019	0.170	[0.682, 1.356]	5.982	41.1	4.56×10^{-7}	Exploratory (secondary)	0.593	False	True	47	139
CK	sex1	-12.206	23.345	[-58.397, 33.986]	-0.523	41.0	0.604	Exploratory (secondary)	0.593	False	True	47	139
CK	SCFL	-3.147	5.492	[-14.014, 7.720]	-0.573	41.1	0.570	Exploratory (secondary)	0.593	False	True	47	139
CK	time_std:condition1	16.050	10.621	[-4.965, 37.065]	1.511	89.4	0.134	Exploratory (secondary)	0.593	False	True	47	139
CK	time_std:condition2	0.648	10.925	[-20.968, 22.264]	0.059	89.7	0.953	Exploratory (secondary)	0.593	False	True	47	139
CRP[a]	(Intercept)	-0.247	0.152	[-0.547, 0.054]	-1.626	41.1	0.112	Grand mean (intercept)	0.809	False	True	47	139
CRP[a]	time_std	-0.057	0.015	[-0.087, -0.027]	-3.742	89.2	3.22×10^{-4}	Exploratory (secondary)	0.809	False	True	47	139
CRP[a]	condition1	0.142	0.079	[-0.015, 0.299]	1.791	41.0	0.081	Exploratory (secondary)	0.809	False	True	47	139
CRP[a]	condition2	-0.109	0.081	[-0.270, 0.052]	-1.334	41.4	0.189	Exploratory (secondary)	0.809	False	True	47	139
CRP[a]	log_CRP_baseline	0.741	0.064	[0.614, 0.868]	11.536	41.1	1.83×10^{-14}	Exploratory (secondary)	0.809	False	True	47	139
CRP[a]	sex1	-0.083	0.072	[-0.224, 0.059]	-1.151	41.1	0.256	Exploratory (secondary)	0.809	False	True	47	139
CRP[a]	SCFL	0.010	0.017	[-0.025, 0.044]	0.558	41.1	0.580	Exploratory (secondary)	0.809	False	True	47	139
CRP[a]	time_std:condition1	0.026	0.021	[-0.016, 0.068]	1.228	89.2	0.223	Exploratory (secondary)	0.809	False	True	47	139
CRP[a]	time_std:condition2	-0.073	0.022	[-0.116, -0.029]	-3.313	89.3	0.001	Exploratory (secondary)	0.809	False	True	47	139
ESR	(Intercept)	1.979	1.109	[-0.217, 4.175]	1.784	40.5	0.082	Grand mean (intercept)	0.476	False	True	46	135
ESR	time_std	-0.450	0.205	[-0.857, -0.044]	-2.193	85.6	0.031	Exploratory (secondary)	0.476	False	True	46	135
ESR	condition1	0.508	0.555	[-0.591, 1.607]	0.915	41.3	0.365	Exploratory (secondary)	0.476	False	True	46	135

Continued on next page

Table 1: **S1 Table.** Full linear mixed-effects model output and diagnostics (continued).

**Outcome**	**Fixed effect term**	**Est.**	**SE**	**95% CI**	t	**df**	p	**Note**	**ICC**	**Sing.**	**Conv.**	n_{subj}	n_{obs}
ESR	condition2	0.050	0.576	[-1.089, 1.190]	0.087	41.0	0.931	Exploratory (secondary)	0.476	False	True	46	135
ESR	ESR_baseline	0.668	0.079	[0.511, 0.825]	8.424	40.2	2.03×10^{-10}	Exploratory (secondary)	0.476	False	True	46	135
ESR	sex1	-0.630	0.519	[-1.657, 0.397]	-1.214	40.7	0.232	Exploratory (secondary)	0.476	False	True	46	135
ESR	SCFL	-0.019	0.119	[-0.255, 0.217]	-0.162	41.0	0.872	Exploratory (secondary)	0.476	False	True	46	135
ESR	time_std:condition1	-0.192	0.300	[-0.787, 0.402]	-0.640	86.2	0.524	Exploratory (secondary)	0.476	False	True	46	135
ESR	time_std:condition2	0.177	0.288	[-0.392, 0.747]	0.616	85.3	0.539	Exploratory (secondary)	0.476	False	True	46	135
MT	(Intercept)	0.420	0.253	[-0.081, 0.921]	1.658	42.0	0.105	Grand mean (intercept)	0.522	False	True	48	144
MT	time_std	-0.008	0.017	[-0.042, 0.025]	-0.479	93.0	0.633	Exploratory (secondary)	0.522	False	True	48	144
MT	condition1	-0.006	0.051	[-0.107, 0.094]	-0.120	42.0	0.905	Exploratory (secondary)	0.522	False	True	48	144
MT	condition2	-0.061	0.050	[-0.160, 0.038]	-1.216	42.0	0.231	Exploratory (secondary)	0.522	False	True	48	144
MT	MT_baseline	0.904	0.056	[0.792, 1.015]	15.998	42.0	1.78×10^{-19}	Exploratory (secondary)	0.522	False	True	48	144
MT	sex1	0.002	0.047	[-0.092, 0.095]	0.032	42.0	0.975	Exploratory (secondary)	0.522	False	True	48	144
MT	SCFL	0.005	0.011	[-0.017, 0.027]	0.454	42.0	0.652	Exploratory (secondary)	0.522	False	True	48	144
MT	time_std:condition1	-0.019	0.024	[-0.066, 0.029]	-0.785	93.0	0.435	Exploratory (secondary)	0.522	False	True	48	144

MT	time_std:condition2	-0.002	0.024	[-0.049, 0.045]	-0.089	93.0	0.929	Exploratory (secondary)	0.522	False	True	48	144

Notes. Fixed-effect estimates are from the selected linear mixed-effects model (LMM, REML) for each outcome; all outcomes used a linear time specification as determined by model selection (LRT + AIC). Test statistics are Wald t-statistics with Satterthwaite degrees of freedom. 95% confidence intervals are Wald confidence intervals derived from the fitted model. All models fitted with sum-to-zero contrasts (`contr.sum`): **condition1** = CG vs. grand mean; **condition2** = ICG vs. grand mean; DCG effect = $-(\text{condition1} + \text{condition2})$. **sex1** = male vs. grand mean. CRP was modelled on the natural log scale; **log_CRP_baseline** is the log-transformed baseline covariate. All p-values in this table are uncorrected (raw). Multiplicity correction (Holm, within primary outcome) was applied separately to post-hoc pairwise contrasts from *emmeans* and is reported in the main results; it was not applied to these fixed-effect coefficients. ICC is the intraclass correlation coefficient from the random-intercept structure. *Sing.* = singular random-effects fit (all False in current models). *Conv.* = model convergence confirmed. "–" = not applicable.

[a] CRP modelled on the natural log scale. Estimates and SEs are in log units (ln mg/L). The baseline covariate `log_CRP_baseline` is likewise log-transformed.
