# Supplementary material for: Effects of Immediate and Delayed Repeated Cold Exposure After Physical Exertion: A Randomized Controlled Trial
Source: Int J Environ Res Public Health. 2026 Jul 9;23(7):887. doi: 10.3390/ijerph23070887 (PMC13410065; doi:10.3390/ijerph23070887)
Supplement: Supplementary file 1 [file ijerph-23-00887-s001.zip › FileS2_CIP.pdf]

# Clinical Investigation Plan (CIP)

EFFECTS OF IMMEDIATE AND DELAYED REPEATED COLD EXPOSURE AFTER PHYSICAL EXERTION: A RANDOMISED CONTROLLED TRIAL

| | |
|---|---|
| Type of investigation: | Clinical investigation concerning medical devices (MD). |
| Categorisation: | Category according to Art 6 ClinO-MD A1 |
| Registration: | NCT06813690 |
| Identifier: | 2024-D0116 |
| Principal Investigator and Sponsor, or Sponsor-Investigator: | Dr. Ron Clijsen<br>University of Applied Sciences of Southern Switzerland SUPSI<br>Rehabilitation and Exercise Science Laboratory (RES Lab)<br>Weststrasse 8<br>7302 Landquart<br>+41 81 300 01 75<br><br>Thim van der Laan jr<br>Thim van der Laan AG<br>Weststrasse 8<br>7302 Landquart<br>+41 81 300 01 70 |
| Sponsor representative (if the Sponsor is not located in Switzerland) | - |
| Medical Device: | Axanova Cold Hot Pearls Kompressen<br>Basic UDI-DI (GMN): 764011364_PG_04_RF |
| CIP Version and Date: | Version 06 (03.03.2025) |

**CONFIDENTIALITY STATEMENT**

The information contained in this document is confidential and the property of University of Applied Sciences of Southern Switzerland SUPSI, Rehabilitation and Exercise Science Laboratory (RESLab). The information may not - in full or in part - be transmitted, reproduced, published, or disclosed to others than the applicable Competent Ethics Committee(s) and Regulatory Authority(ies) without prior written authorisation from the sponsor except to the extent necessary to obtain informed consent from those who will participate in the Investigation.

Signature Page

| | |
|---|---|
| ID number of the investigation: | 2024-D0116 |
| Title: | Effects of immediate and delayed repeated cold exposure after physical exertion: a randomised controlled trial |

The Sponsor, the Principal Investigator and the Statistician have approved the CIP version [6 (dated 03.03.2025),], and confirm hereby to conduct the investigation according to the CIP, the current version of the World Medical Association Declaration of Helsinki, ISO14155 norm [2024], ICH-GCP as far as applicable, and the local legally applicable requirements.
The Investigator has received the ICF and consider it appropriate for use.

**Sponsor:**

Name: *Thim van der Laan jr.*

Place/Date ______________________ Signature ______________________

**Principal Investigator:**

Name: *Dr. Ron Clijsen*

Place/Date ______________________ Signature ______________________

**TABLE OF CONTENTS**

SYNOPSIS 4
ABBREVATIONS 8
1 BACKGROUND AND RATIONALE 9
2 INVESTIGATION OBJECTIVES AND DESIGN 9
2.1 Hypothesis and primary objective 9
2.2 Primary and secondary endpoints 10
2.3 Investigation design 11
2.4 Investigation intervention 12
3 INVESTIGATION POPULATION AND INVESTIGATION PROCEDURES 12
3.1 Inclusion and exclusion criteria, justification of Investigation population 12
3.2 Recruitment, screening and informed consent procedure 12
3.3 Investigation procedures 13
3.4 Withdrawal and discontinuation 15
4 STATISTICS AND METHODOLOGY 15
4.1. Statistical analysis plan and sample size calculation 15
4.2. Handling of missing data and drop-outs 17
5 SAFETY 18
5.1 Definition and Assessment of (Serious) Adverse Events and other safety related events 18
5.2 Documentation and reporting in Medical Device Category A clinical investigations 19
5.2.1 Foreseeable adverse events and anticipated adverse device effects 20
5.2.2 Reporting of Safety related events 21
5.3 Radiation 21
5.4 Amendments (Art. 15 ClinO-MD) 21
5.5 Notification and reporting upon completion, discontinuation or interruption of the Investigation 22
5.6 Insurance 22
6 FURTHER ASPECTS 22
6.1 Overall ethical considerations 22
6.2 Risk-benefit assessment 23
7. QUALITY CONTROL AND DATA PROTECTION 23
7.1 Quality measures 23
7.2 Data recording and source data 24
7.3 Confidentiality and coding 24
7.4 Retention and destruction of Investigation data and biological material 25
8 MONITORING AND REGISTRATION 25
9. FUNDING / PUBLICATION / DECLARATION OF INTEREST 26
10. REFERENCES 27

## SYNOPSIS

| | |
|---|---|
| **Sponsor / Sponsor-Investigator** | Thim van der Laan AG<br>Thim van der Laan jr.<br>Weststrasse 8, CH-7302 Landquart<br><br>Fachhochschule Südschweiz (SUPSI)<br>Dr. Ron Clijsen<br>Weststrasse 8, CH-7302 Landquart |
| **Title** | Effects of immediate and delayed repeated cold exposure after physical exertion: a randomised controlled trial |
| **Short Title / Invesigation ID** | Local cooling and recovery |
| **Protocol Version and Date** | Version *06 of date (03/03/2025)* |
| **Registration** | ClinicalTrials.gov PRS |
| **Category and Rationale** | Kategorie nach Art 6 ClinO-MD A1.<br>In diese Untersuchung wird untersucht, ob sich wiederholte, lokale Kälteapplikationen positiv auf die Erholung auswirken nach einem Muskelkaterprotokoll. Den Probanden werden weder Medikamente verabreicht, noch werden Gewebeproben entnommen. Es kommen minimal invasive Techniken zum Einsatz (venöse Blutabnahme). |
| **Name of the MD, Unique Device Identification (UDI), name of the manufacturer** | Axanova Cold Hot Pearls Kompressen<br>Basic UDI-DI (GMN): 764011364_PG_04_RF<br>Axanova AG |
| **Stage of development:** | Post-market stage |
| **Background and Rationale** | Sportliche Aktivität ist geprägt von Faktoren der Belastungsart, -dauer und -intensität. Abhängig vom Umfang dieser Faktoren und der damit verbundenen Erholungszeit entstehen Schädigungen an der Muskulatur, Entzündungs- und Ermüdungserscheinungen im Nervensystem. Ebenso kommt es zum Energiesubstratabbau und zur lokalen Schwellung. Daher ist eine schnelle Regeneration nach intensivem Sport umso bedeutsamer geworden. Nach der Metaanalyse von Bleakley et al. 2012 wird Kälte als einen der effektivsten Regenerationsmethode nach sportlicher Aktivität angesehen, um die „Delayed Onset Muscle Soreness“ (DOMS) hinauszuzögern. Die DOMS sind mikroskopisch kleine Risse im Muskelgewebe, die als trainingsinduzierte Muskelschäden bezeichnet werden und zu einem verzögert auftretenden Muskelkater führen können. DOMS erreichen ihren Höhepunkt üblicherweise zwischen 24 und 48 Stunden – teilweise auch bis zu 72 Stunden – nach dem Training und sind gekennzeichnet durch Muskelverkürzung, erhöhte passive Steifigkeit, Schwellung, Abnahme von Kraft und Leistung, lokalisiertem Muskelkater und veränderte Propriozeption.<br>Der physiologische Hintergrund der Kryotherapie basiert auf der Abfuhr von Körperwärme durch die Verringerung der Gewebstemperatur. Diese zeigt sich in einer verringerten Muskelschmerzwahrnehmung, sodass sich der Körper nach dem Training „wacher“ anfühlt und ein geringeres Ermüdungsgefühl verursacht. Darüber hinaus senkt der Körper aufgrund der Kälte die Herzfrequenz und das Herzzeitvolumen und induziert eine Vasokonstriktion. Resultate sind kleinere Gefässdurchmesser, ein reduziertes Auftreten von Ödemen und eine verbesserte Sauerstoffversorgung der Zellen. Um die Kerntemperatur des Körpers aufrechtzuerhalten, steigt zusätzlich der zentrale Stoffwechsel an, was den Transport von Abfallprodukten begünstigt. All diese Effekte könnten in Komposition, die durch körperliche Betätigung verursachten Entzündungen verringern, indem sie den Tod oder die Schädigung hypoxischer Zellen mindern und durch Verringerung der Infiltration von Leukozyten und Monozyten die Schädigung des Sekundärgewebes minimieren. (Bleakley et al. 2012, Hohenauer E et al. 2015, Hubbard et al. 2004, Ostrowski et al 2018). Die postoperative Kryotherapie unter Verwendung von Eisbeuteln, Kühlung oder kontinuierlichen Kryotherapiegeräten reduzierte die Schmerzwerte auf der visuellen Analogskala und den |

| | |
|---|---|
| | Analgetikaverbrauch in etwa bei der Hälfte der im Stand der Technik untersuchten Forschungsstudien, in denen diese Ergebnisse mit der Kontrollgruppe (keine Kryotherapie) verglichen wurden (11 [44 %] von 25 Studien zu Schmerzen und 11 [48 %] von 23 Studien zu Opioiden). Allerdings wurde seltener über einen Effekt bei der Vergrösserung des Bewegungsumfangs (3 [19 %] von 16) oder der Verringerung der Schwellung (2 [22 %] von 9) berichtet. (Kunkle et al. 2021). Die hier induzierte Untersuchung soll neue Daten vor allem bei der Reduktion von Schwellung und Entzündungswerten liefern und die Reduktion von Schmerzen durch Kälteanwendung des Axanova Cold Hot Pearls Maxi Pack bestätigen. |
| **Risk / Benefit Assessment** | In dieser Untersuchung kann das Risiko als minimal eingestuft werden. Das Produkt wird so appliziert, wie vom Hersteller empfohlen. Das Muskelkaterprotokoll ist ein Protokoll, dass bereits in der wissenschaftlichen Literatur beschrieben wurde. |
| **Objective(s)** | Ziel dieser Untersuchung ist es zu untersuchen, wie sich lokale, wiederholte Kälteapplikationen auf die Erholungsphase auswirken nach einem Muskelkaterprotokoll.<br>Die Erholung wird in 24 Stunden Abständen (bis zu 72 Stunden nach dem Muskelkaterprotokoll) untersucht. |
| **Endpoint(s)** | Primäre Endpunkte:<br>• Muskelkaterbeschwerden (VAS 0 – 10 cm)<br>• Entzündungswerte (Blutsenkgeschwindigkeit, C-reaktives Protein, Kreatin-kinase)<br>• Muskelschwellung (in cm mittels Ultraschall)<br>• Maximale willkürliche isometrische Oberschenkelkontraktion (in kg)<br>Sekundäre Endpunkte<br>• Oberflächentemperatur der Haut (in °C)<br>• Thermische Wahrnehmung |
| **Investigation Design** | Randomisierte, kontrollierte Studie |
| **Statistical Considerations** | Repeated measure ANOVA, n=45 Probanden |
| **Inclusion- / Exclusion Criteria** | Einschlusskriterien<br>• Junge, gesunde Erwachsene im Alter zwischen 18 und 30 Jahren<br>• Keine operativen Eingriffe am muskuloskeletalen System im Rumpfbereich und an den unteren Extremitäten<br>Ausschlusskriterien<br>• Aktuelle Schmerzzustände: Probanden mit akuten Schmerzzuständen.<br>• Aktuelle Entzündungszustände: Vorliegen von Entzündungen oder bekannten entzündlichen Erkrankungen.<br>• Medikamenteneinnahme (exkl. Antikonzeptiva): Probanden, die Medikamente einnehmen, mit Ausnahme von Antikonzeptiva.<br>• Schwangere Probandinnen: Frauen, die schwanger sind oder stillen.<br>• Leistungssportler: Probanden, die Leistungssport betreiben.<br>• Kinder/Jugendliche: Probanden unter 18 Jahren oder über 30 Jahre.<br>• Nicht intakte Hautzustände (z. B. Schuppenflechte): Hauterkrankungen, wie Schuppenflechte oder andere nicht intakte Hautzustände.<br>• Bekannte Durchblutungsstörungen: Vorhandensein bekannter Durchblutungsstörungen.<br>• Kälteallergie (Raynaud-Syndrom): Allergie gegenüber Kälte oder das Vorliegen des Raynaud-Syndroms.<br>• Frühere Operationen am Oberkörper oder den Beinen: Operationen in den untersuchten Körperbereichen.<br>• Gestörte Wahrnehmung am Oberschenkel: Störungen der Temperatur- oder Berührungsempfindung im Oberschenkelbereich.<br>• Diagnostizierte Krankheiten: Vorhandensein von diagnostizierten Krankheiten, die die Teilnahme an der Untersuchung beeinflussen könnten.<br>• Rauchen: Wenn der Proband raucht. |
| **Number of Participants with Rationale** | Die Untersuchung ist mit 3 Studienarmen geplant. Der durchschnittliche Initiale VAS Score nach Schmerzinduktion variiert je nach Untersuchung und liegt als Näherung bei einem |

| | |
|---|---|
| | Ausgangswert von 4,73 ± 2,39. Eine Reduktion des VAS-Scores nach Kälte Applikation wurde mit durchschnittlich 47% angenommen. Nach Berücksichtigung der Dropout-Rate von 10% und Aufsplittung in 3 randomisierte Gruppe wird eine Patientenzahl von 15 Probanden pro Studienarm als statistisch angemessen betrachtet. Die Ergebnisse wurden mit der Software PASS 2023 Version 23.0.1 ermittelt. |
| **Intervention** | Zur Überprüfung wird das Produkt "Cold Hot Pearls Maxi Pack" der Firma Axanova AG verwendet. Dieses Produkt verfügt über eine Gelperlenfüllung, welche für Kälte und Wärmeapplikationen verwendet werden kann. Die Kältekompressen müssen gemäss Hersteller mindestens 1 Stunde vorgängig in einem Tiefkühler gelagert werden, bevor sie gebraucht werden können. Im Anschluss kann der Kältepack auf die betreffende Stelle gelegt werden auf der Vliesseite, maximal jedoch 20 Minuten an derselben Stelle.<br>In unserer Untersuchung sind 2 Kühlgruppen vorhanden:<br>Die erste Gruppe beginnt mit der Kühlung sofort nach dem Muskelkaterprotokoll.<br>Die zweite Gruppe beginnt mit der Kühlung 24 Stunden nach dem Muskelkaterprotokoll. |
| **Control Intervention** | Die Kontrollgruppe erhält keine Intervention nach dem Muskelkaterprotokoll. |
| **Investigation procedures** | **Probandenrekrutierung**<br>Die ProbandInnen werden an der Hochschule für Physiotherapie THIM in Landquart und der Fachhochschule Südschweiz in Landquart rekrutiert.<br>Die Probandeninformation sowie die Einwilligungserklärung werden den ProbandInnen abgegeben. In der Probandeninformation befindet sich eine Checkliste, welche die ProbandInnen ausfüllen müssen, um die Ein- und Ausschlusskriterien zu prüfen.<br>Wenn die ProbandInnen mit den Informationen einverstanden sind, die Checkliste ausgefüllt haben und die Einwilligung unterzeichnet haben, werden sie aufgeboten, um einen Termin für die Messung zu vereinbaren.<br>**Untersuchungsprotokoll**<br>Sobald die unterschriebene Probandeneinwilligung vorliegt und ein Termin vereinbart wurde, wird wie folgt vorgegangen. Die anthropometrischen Daten der Probanden werden erhoben und die Gruppenrandomisierung durchgeführt. Im Anschluss werden die Baselinemessungen durchgeführt und die Messung der Hauttemperatur gestartet. Danach wird das Muskelkaterprotokoll (6 x 12 bis 15 einbeinige Kniestrecker-Protokoll mit 2 min Pause zwischen den Sets) absolviert. Im Anschluss erhält die Kühlgruppe A die erste 20 minütige Kühlung der Oberschenkelgruppe.<br>Die Kühlgruppe B sowie die Kontrollgruppe verlassen das Labor 20 Minuten nach dem Muskelkaterprotokoll. Die Erhebung der Hauttemperatur wird innerhalb aller Gruppen 20 min nach dem Muskelkaterprotokoll gestoppt.<br>Die Kühlgruppe B beginnt mit der Kühlung der Oberschenkelmuskulatur 24 Stunden nach dem Muskelkaterprotokoll. Beide Kühlgruppen führen die Kühlung der Oberschenkelmuskulatur 3 x / Tag für die Dauer der Datenerhebung (bis hin zu 72 Stunden nach dem Muskelkaterprotokoll) durch.<br>Im Abstand von 24, 48 und 72 Stunden werden erneut die primären Endpunkte der Untersuchung erhoben. Diese umfassen: Muskelkaterbeschwerden, Entzündungswerte, Muskelschwellung, Maximale willkürliche isometrische Kontraktion der Oberschenkelmuskulatur.<br>**Messung der Muskelkaterbeschwerden**<br>Die Höhe der ventral empfundenen Muskelkatersymptome wird mittels einer VAS-Skala (0 – 10 cm) gemessen. Die Probanden bewerten den empfundenen Muskelkater an beiden ventralen Oberschenkeln in einer 90° Knieflexions-position. Die Messung findet während der Baselinemessung und, 24 Stunden, 48 Stunden und 72 Stunden nach der Intervention statt.<br>**Entzündungswerte**<br>Die Entzündungswerte (Blutsenkgeschwindigkeit, C- reaktives Protein, Kreatin-kinase) werden mittels einer venösen Blutabnahme bestimmt. Die Messung findet während der Baselinemessung und, 24 Stunden, 48 Stunden und 72 Stunden nach der Intervention statt.<br>**Muskelschwellung**<br>Die Messung der Muskelschwellung des Quadriceps femoris Muskel erfolgt mittels Ultraschallmessung. Die Messung wird in Ruhelage durchgeführt und ein Querschnitt des Muskels aufgenommen. Die Messung findet während der Baselinemessung und, 24 Stunden, 48 Stunden und 72 Stunden nach der Intervention statt. |

| | |
|---|---|
| | **Messung der maximalen willkürlichen isometrischen Oberschenkelmuskelkontraktion (MVIC)**<br>MVIC wird auf einem Ergometer Stuhl für biomechanische Messungen durchgeführt und in kg angegeben. Die Messung findet während der Baselinemessung und, 24 Stunden, 48 Stunden und 72 Stunden nach der Intervention statt.<br>**Oberflächentemperatur**<br>Die Oberflächentemperatur wird mittels iButton System gemessen. Diese Sensoren werden auf die Haut geklebt für die Dauer der Messung. Die Knopfsensoren speichern die aktuelle Temperaturangaben und können im Anschluss ausgelesen werden über einen Computer. Diese Messungen wird während der Baseline-erhebung gestartet, für die Dauer des Muskelkaterprotokolles, und bis hin 20 min nach dem Muskelkaterprotokoll.<br>**Erfassung der thermischen Wahrnehmung**<br>Die thermische Wahrnehmung der Teilnehmenden wird unmittelbar nach der Kälteeinwirkung mithilfe der standardisierten Thermal Perception Skala (-4 bis +4) erfasst. Diese Skala reicht von -4 ("extrem kalt") bis +4 ("extrem warm"), wobei 0 als neutral gilt. Die Bewertung erfolgt als subjektive Selbsteinschätzung, um die individuelle Temperaturempfindung zu dokumentieren. Die Teilnehmenden werden vorab über die Skala informiert. Da es sich um eine rein subjektive Einschätzung handelt, besteht kein Risiko für die Teilnehmenden. |
| **Investigation Duration and Schedule** | Geplanter Start der Messungen: 01/2025<br>Geplanter Ende der Messungen: 07/2025 |
| **Investigator(s)** | Dr. Ron Clijsen<br>Dr. Erich Hohenauer<br>Vanessa Wellauer<br><br>Kontakt / Studienleiter<br>Clijsen Ron, PhD Fachhochschule Südschweiz Physiotherapie Graubünden Weststrasse 8, 7302 Landquart +41 81 300 01 75 ron.clijsen@supsi.ch |
| **Investigation Center(s)** | Fachhochschule Südschweiz Physiotherapie Graubünden Rehabilitation Research Laboratory RESlab SUPSI Landquart, Weststrasse 8 7302 Landquart |
| **Data privacy** | Die Daten sind nur dem Studienleiter (Ron Clijsen) sowie Personen der KEK (bei allfälligen Qualitätskontrollen vor Ort) zugänglich. Die Daten werden nummerisch codiert (001, 002, 003, etc.). Nur der Studienleiter kann den Code auflösen. Alle Daten werden im Labor der Fachhochschule Südschweiz in Landquart gelagert. |
| **Ethical consideration** | SportlerInnen könnten von diesen Ergebnissen profitieren, da sie Informationen erhalten, wie sie ihre sportliche Leistungsfähigkeit bzw. Erholung mittels Kälteapplikationen nach Belastung erhöhen könnten. Die methodologische Vorgehensweise orientiert sich an der aktuellen wissenschaftlichen Literatur. Das Risiko kann als minimal angesehen werden, da das Produkt so verwendet wird wie vorgeschrieben. |
| **Compliance statement** | This investigation will be conducted in full compliance with the CIP, the current version of the Declaration of Helsinki, ISO 14155 [2024], ICH-GCP (as far as applicable) as well as all national legal and regulatory requirements. |

**ABBREVATIONS**

| | |
|---|---|
| *AE* | *Adverse Event* |
| *AIP* | *Azure Information Protection* |
| *ASR* | *Annual Safety Repot* |
| *BASEC* | *Business Administration System for Ethical Committees* |
| *ClinO* | *Ordinance on Clinical Trials in Human Research (in German: KlinV, in French: OClin, in Italian: OSRUm)* |
| *CRF* | *Case Report Form* |
| *CTCAE* | *Common Terminology Criteria for Adverse Events* |
| *DOMS* | *Delayed Onset Muscle Soreness* |
| *eCRF* | *electronic Case Report Form* |
| *FADP* | *Federal Act on Data Protection (in German: DSG, in French: LPD, in Italian: LPD)* |
| *FOPH* | *Federal Office of Public Health* |
| *GCP* | *Good Clinical Practice* |
| *HRA* | *Human Research Act (in German: HFG, in French: LRH, in Italian: LRUm)* |
| *ICH* | *International Conference on Harmonisation* |
| *ISO* | *International Organisation for Standardisation* |
| *MDR* | *Medical Device Regulation* |
| *MVIC* | *Maximum Voluntary Isometric Contraction* |
| *SAE* | *Serious Adverse Event* |
| *SDV* | *Source Data Verification* |
| *SSL* | *Secure Sockets Layer* |
| *SUE* | *Serious Unexpected Events* |
| *TLS* | *Transport Layer Security* |
| *UAE* | *Unanticipated Adverse Event* |

# 1 BACKGROUND AND RATIONALE

Sportliche Aktivität ist geprägt von Faktoren der Belastungsart, -dauer und -intensität. Abhängig vom Umfang dieser Faktoren und der damit verbundenen Erholungszeit entstehen Schädigungen an der Muskulatur, Entzündungs- und Ermüdungserscheinungen im Nervensystem, ebenso kommt es zum Energiesubstratabbau und zur lokalen Schwellung. Daher ist eine schnelle Regeneration nach intensivem Sport umso bedeutsamer geworden. Nach der Metaanalyse von Bleakley et al. 2012 wird Kälte als einer der effektivsten Regenerationsmethode nach sportlicher Aktivität angesehen, um die „Delayed Onset Muscle Soreness" (DOMS) hinauszuzögern. Die DOMS sind mikroskopisch kleine Risse im Muskelgewebe, die als trainingsinduzierte Muskelschäden bezeichnet werden und zu einem verzögert auftretenden Muskelkater führen können. DOMS erreichen ihren Höhepunkt üblicherweise zwischen 24 und 48 Stunden – teilweise auch bis zu 72 Stunden – nach dem Training und sind gekennzeichnet durch Muskelverkürzung, erhöhte passive Steifigkeit, Schwellung, Abnahme von Kraft und Leistung, lokalisierten Muskelkater und veränderte Propriozeption.

Der physiologische Hintergrund der Kryotherapie basiert auf der Abfuhr von Körperwärme durch die Verringerung der Gewebstemperatur. Diese zeigt sich in einer verringerten Muskelschmerzwahrnehmung, sodass sich der Körper nach dem Training „wacher" anfühlt und ein geringeres Ermüdungsgefühl verursacht. Darüber hinaus senkt der Körper aufgrund der Kälte die Herzfrequenz und das Herzzeitvolumen und induziert eine Vasokonstriktion. Resultate sind kleinere Gefässdurchmesser, ein reduziertes Auftreten von Ödemen und eine verbesserte Sauerstoffversorgung der Zellen. Um die Kerntemperatur des Körpers aufrechtzuerhalten, steigt zusätzlich der zentrale Stoffwechsel an, was den Transport von Abfallprodukten begünstigt. All diese Effekte könnten in Komposition, die durch körperliche Betätigung verursachten Entzündungen verringern, indem sie den Tod oder die Schädigung hypoxischer Zellen mindern und durch Verringerung der Infiltration von Leukozyten und Monozyten die Schädigung des Sekundärgewebes minimieren. (Bleakley et al. 2012, Hohenauer E et al. 2015, Hubbard et al. 2004, Ostrowski et al 2018). Die postoperative Kryotherapie unter Verwendung von Eisbeuteln, Kühlung oder kontinuierlichen Kryotherapiegeräten reduzierte die Schmerzwerte auf der visuellen Analogskala und den Analgetika Verbrauch in etwa bei der Hälfte der im Stand der Technik untersuchten Forschungsstudien, in denen diese Ergebnisse mit der Kontrollgruppe (keine Kryotherapie) verglichen wurden (11 [44 %] von 25 Studien zu Schmerzen und 11 [48 %] von 23 Studien zu Opioiden). Allerdings wurde seltener über einen Effekt bei der Vergrösserung des Bewegungsumfangs (3 [19 %] von 16) oder der Verringerung der Schwellung (2 [22 %] von 9) berichtet. (Kunkle et al. 2021). Die hier induzierte Untersuchung soll neue Daten vor allem bei der Reduktion von Schwellung und Entzündungswerten liefern und die Reduktion von Schmerzen durch Kälteanwendung des Axanova Cold Hot Pearls Maxi Pack bestätigen.

# 2 INVESTIGATION OBJECTIVES AND DESIGN

## 2.1 Hypothesis and primary objective

Das Ziel dieser Untersuchung ist es zu untersuchen, ob wiederholte, lokale Kälteapplikationen einen positiven Einfluss haben auf die Erholung im Vergleich zu keiner Intervention. Weiters soll untersucht werden, ob Kühlungen, welche direkt nach der Belastung durchgeführt haben die Erholung positiver beeinflussen, im Vergleich zu Kühlungen, welche 24 Stunden nach der Belastung begonnen haben. Die Erholungsfähigkeit wird anhand folgender Parameter bestimmt:

Muskelkaterbeschwerden, Entzündungswerte, Muskelschwellung und maximal willkürliche isometrische Kontraktion der Oberschenkelmuskulatur.

- $H_{0A}$: Es gibt keinen signifikanten Unterschied zwischen wiederholten Kälteapplikationen und der Kontrollintervention in Bezug auf die Erholungsfähigkeit.
- $H_{1A}$: Es gibt einen signifikanten Unterschied zwischen wiederholten Kälteapplikationen und der Kontrollintervention in Bezug auf die Erholungsfähigkeit.

- $H_{0A}$: Es gibt keinen signifikanten Unterschied zwischen sofort durchgeführten Kälteapplikationen und verzögerten Kälteapplikationen in Bezug auf die Erholungsfähigkeit.

$H_{1A}$: Es gibt einen signifikanten Unterschied zwischen sofort durchgeführten Kälteapplikationen und verzögerten Kälteapplikationen in Bezug auf die Erholungsfähigkeit.

## 2.2 Primary and secondary endpoints

### Primäre Endpunkte

Muskelkaterbeschwerden

Zur quantitativen Erfassung von Muskelkaterbeschwerden wird eine visuelle Analogskala (VAS) verwendet. Die Muskelkaterangaben der ProbandInnen werden an beiden ventralen Oberschenkeln in 90° Knieflexion innerhalb von 2 Sekunden gemessen. Die Teilnehmer führen eine Kniebeuge in einem 90-Grad-Winkel aus und nutzen eine handgehaltene Gleitskala, um einen Wert zwischen 0 und 10 cm zu wählen, wobei 0 für keine Beschwerden und 10 für maximale Beschwerden steht. Diese Skala ist ohne sichtbare numerische Indikatoren gestaltet, um Wiederholungsbias zu minimieren. Die Messungen erfolgen während der Baseline sowie 24, 48 und 72 Stunden nach der Intervention. Das Untersuchungspersonal erfasst den gewählten Wert in Zentimeter, um die Höhe der ventral empfundenen Muskelkatersymptome zu dokumentieren.

Entzündungswerte

Die allgemeinen Entzündungswerte werden anhand von 3 Parametern bestimmt. Diese sind die Blutsenkgeschwindigkeit, das C-reaktive Protein sowie die Kreatin-Kinase. Diese Parameter werden anhand einer venöse Blutabnahme bestimmt.

Muskelschwellung

Die Schwellung der ventralen Oberschenkelmuskulatur wird mittels Ultraschalldiagnositk durchgeführt. Die ProbanInnen befinden sich in liegender Position. Im Anschluss wird ein Querschnittsbild der Oberschenkelmuskulatur erstellt und die Distanz vom Oberschenkelknochen bis hin zur äusseren Grenze der Muskulatur gemessen.

Maximale willkürliche isometrische Muskelkontraktion

Dieser Parameter wird anhand eines Ergometer Stuhles in 90° Hüftflexion gemessen. Die ProbandInnen müssen während 3 Sekunden versuchen, ihren Oberschenkel so stark als möglich zu strecken. Der maximale Ausschlag wird verwendet, um die maximale willkürliche isometrische Muskelkontraktion zu bestimmen.

### Sekundäre Endpunkte

Oberflächige Hauttemperatur

Die Hauttemperatur der Oberschenkel wird mittels dem iButton System gemessen. Die Sensoren werden auf die Haut aufgeklebt und messen kontinuierliche die Temperatur der Haut. Im

Anschluss werden die Daten der Sensoren auf einem Computer ausgelesen. Diese Messung wird während der Baseline-erhebung gestartet, für die Dauer des Muskelkaterprotokolles, und bis hin 20 min nach dem Muskelkaterprotokoll.

Thermische Wahrnehmung

Die thermische Wahrnehmung der Teilnehmenden wird unmittelbar nach der Kälteeinwirkung mithilfe der standardisierten Thermal Perception Skala (-4 bis +4) erfasst. Diese Skala reicht von -4 ("extrem kalt") bis +4 ("extrem warm"), wobei 0 als neutral gilt. Die Bewertung erfolgt als subjektive Selbsteinschätzung, um die individuelle Temperaturempfindung zu dokumentieren. Die Teilnehmenden werden vorab über die Skala informiert. Da es sich um eine rein subjektive Einschätzung handelt, besteht kein Risiko für die Teilnehmenden.

### 2.3 Investigation design

Diese Untersuchung ist eine monozentrische, randomisierte, kontrollierte Studie mit 3 Armen. Nachdem die ProbandInnen zur Untersuchung zugelassen wurden, werden sie in eine der 3 Gruppen eingeteilt.

Gruppe A: sofortige Kühlung nach dem Muskelkaterprotokoll

Gruppe B: verzögerte Kühlung nach dem Muskelkaterprotokoll (24 Stunden verzögert)

Gruppe C: Kontrollgruppe

Es handelt sich bei dieser Untersuchung um einen klinischen Versuch der Risikokategorie A.

**Gruppe A: sofortige Kühlung**

In dieser Gruppe startet die Kühlung sofort nach dem Muskelkaterprotokoll. Die Kühlung wird 3 x /Tag sowohl am Tag des Muskelkaterprotokolls als auch für die Dauer von 72 Stunden durchgeführt. Beide Oberschenkel werden mit dem "Cold Hot Maxi Pack" der Firma Axanova gekühlt für die Dauer von 20 min.

Kühlprotokoll: Tag 1 (Muskelkaterprotokoll, 3x/Tag), Tag 2 (3x/Tag), Tag 3 (3x/Tag), Tag 4 (3x/Tag)

**Gruppe B: verzögerte Kühlung**

In dieser Gruppe startet die Kühlung 24 Stunden nach dem Muskelkaterprotokoll. Die Kühlung wird ebenfalls 3 x /Tag für die Dauer von 72 Stunden durchgeführt. Beide Oberschenkel werden mit dem "Cold Hot Maxi Pack" der Firma Axanova gekühlt für die Dauer von 20 min.

Kühlprotokoll: Tag 1 (Muskelkaterprotokoll, keine Kühlung), Tag 2 (3x/Tag), Tag 3 (3x/Tag), Tag 4 (3x/Tag).

**Gruppe C: Kontrollgruppe**

Diese Gruppe erhält nach dem Muskelkaterprotokoll und werden der gesamten Periode danach keine Kühlintervention, noch irgendeine andere Intervention.

### Muskelkaterprotokoll

Um Muskelkater hervorzurufen, führen die Probanden einseitige, 6 mal 12 – 15 Kniestrecker auf einem Ergometerstuhl durch. Sie strecken und beugen das Knie so weit wie möglich. Sie wiederholten die Übung nach 2 Minuten Pause noch 5-mal (insgesamt 6 Durchgänge). Die Bewegungsabläufe werden beobachtet und wenn nötig korrigiert. Ein vergleichbares Protokoll wurde bereits erfolgreich in der Literatur verwendet, um Muskelkater zu induzieren (Ruas et al 2022, Comparison between eccentric-only and coupled concentric-eccentric contractions for neuromuscular fatigue and muscle damage). Um die Aussagekraft der Untersuchung zu erhöhen, werden die ProbandInnen gebeten, keine zusätzlichen Interventionen werden der experimentellen Phase durchzuführen.

### 2.4 Investigation intervention

Die wiederholten Kälteapplikationen werden von den ProbandInnen selbstständig zu Hause durchgeführt. Dafür bekommen die ProbandInnen 6 Kältepackungen mit nach Hause (CE-zertifizierte Axanova Cold Hot Pearls Maxi Pack, Axanova AG). Pro Oberschenkel wird eine Kältepackung appliziert (2 Packungen pro Kühlsession). Die Packungen müssen 1 Stunde vor dem Gebrauch in einer Gefriertruhe/Gefrierfach platziert werden. Nachdem die Mindestkühldauer abgelaufen ist, werden die Kältepackungen für 20 min auf die Oberschenkel mit der Vliesseite nach unten (Hautkontakt) aufgelegt. Zwischen den Kälteapplikationen müssen mindestens 4 Stunden liegen. Die Kältepackungen dürfen nur auf die Haut aufgelegt werden, ohne jeglichen, zusätzlichen Druck. Die 20-minütige Kälteapplikation wird vom Hersteller Axanova so beschrieben. Wir ändern die Applikationsdauer sowie Form nicht ab. Die Applikationen 24, 48 und 72 Stunden nach dem Muskelkaterprotokoll werden von den Probanden zu Hause durchgeführt und mit einem Foto dokumentiert. Die Studienteilnehmer laden diese Bilder über einen sicheren, passwortgeschützten Link direkt in einen dafür vorgesehenen, Cloud-basierten Ordner hoch. Dabei wird sichergestellt, dass die Daten verschlüsselt übertragen und gespeichert werden. Wenn die Intervention im Labor durch das Studienpersonal durchgeführt wird, wird die Temperatur jeder Kältepackung mittels Infrarot-Thermometer überprüft, um mögliche Temperaturunterschiede zu dokumentieren. Diese Messung dient der Erfassung potenzieller Variationen in der Kühlwirkung, ohne die Anwendung der Kältepackung zu beeinflussen.

## 3 INVESTIGATION POPULATION AND INVESTIGATION PROCEDURES

### 3.1 Inclusion and exclusion criteria, justification of Investigation population

Für diese Untersuchung werden n=45 ProbandInnen rekrutiert

**Einschlusskriterien**

- Junge, gesunde Erwachsene im Alter zwischen 18 und 30 Jahren
- Keine operativen Eingriffe am muskuloskelettalen System im Rumpfbereich und an den unteren Extremitäten

**Ausschlusskriterien**

- Aktuelle Schmerzzustände
- Aktuelle Entzündungszustände
- Medikamenteneinnahme (exkl. Antikonzeptiva)
- Schwangere Probandinnen
- Leistungssportler
- Kinder/Jugendliche
- Nicht intakte Hautzustände (z.B. Schuppenflechte)
- Bekannte Durchblutungsstörungen
- Kälteallergie (Raynaud-Syndrom)

### 3.2 Recruitment, screening and informed consent procedure

Die Teilnehmer werden über die Websites der Physiotherapieschulen Thim van der Laan (www.physioschule.ch) und der Fachhochschule Südschweiz SUPSI (www.supsi-landquart.ch) rekrutiert. Ein elektronisches Inserat wird auf den Bildschirmen im Gebäude der Thim van der Laan AG in Landquart aufgeschaltet. Die Anzeigentexte richten sich nach den Richtlinien der Ethikkommission des Kantons Zürich.

Der Erstkontakt erfolgt direkt beim Studienleiter in Forschungslabor der Fachhochschule Südschweiz. Beim Erstkontakt werden die potentiellen Teilnehmer über den Ablauf und die Risiken der Untersuchung sowie über die Bedingungen und die Höhe der Entschädigung informiert.

Der Studienleiter erläutern jedem Teilnehmer die Art der Untersuchung, ihren Zweck, die damit verbundenen Verfahren, die voraussichtliche Dauer, die möglichen Risiken und Vorteile sowie etwaige Unannehmlichkeiten, die sie mit sich bringen kann. Jeder Teilnehmer wird darüber informiert, dass die Teilnahme an der Untersuchung freiwillig ist und dass er oder sie jederzeit von der Untersuchung zurücktreten kann und dass der Rücktritt von der Einwilligung keine Auswirkungen auf seine spätere Betreuung hat.

Der Studienleiter händigt im Anschluss die Probandeninformation sowie die Einwilligungserklärung aus. In der Probandeninformation befindet sich eine Checkliste, welche die potentiellen ProbandInnen ausfüllen müssen. In dieser Checkliste wird überprüft, ob die potentiellen ProbandInnen geeignet sind für die Untersuchung. Die Probandinnen wird mitgeteilt, dass sie sich jederzeit melden können, wenn fragen auftauchen.

Die ausgefüllte Checkliste und das unterschriebene Einwilligungsformular werden dem Studienleiter ausgehändigt und von ihm geprüft. Wenn alle Ein-Ausschlusskriterien überprüft, und die Einwilligungserklärung unterzeichnet und der/die ProbandIn keine Fragen mehr hat, ist er/sie zur Untersuchung zugelassen. Eine Kopie der unterzeichneten Einverständniserklärung wird dem Studienteilnehmer ausgehändigt. Die Einverständniserklärung wird als Teil der Studienunterlagen aufbewahrt. Das Original der unterzeichneten Einverständniserklärung wird als Studiendokument in einem verschlossenen Aktenschrank aufbewahrt.

Nach erfolgreichem Abschluss der Intervention erhalten die Teilnehmer eine Vergütung von 50 Schweizer Franken.

### 3.3 Investigation procedures

**Investigation duration:**
Vorbehaltlich der Genehmigung durch die EK im Jahr 2024.
Rekrutierungszeitraum: Januar 2025
Durchführung der Untersuchung: Februar - März 2025
Statistik und Schreiben des Artikels: April – Juli 2025

**Individual Investigation duration for each participant**

| Phase | Inhalt | Zeit |
|---|---|---|
| Rekrutierung | Aushändigen Patienteninformation & Einwilligung, Erklärung des Experiments | 30 min |
| Experiment Tag 1 | Anthropometrie, Baselinemessung, Muskelkaterprotokoll (Kühlung: Gruppe A) | 60 min |
| Experiment Tag 2 | Folgemessung 24 Stunden (Kühlung: Gruppe A und Gruppe B) | 30 min |
| Experiment Tag 3 | Folgemessung 48 Stunden (Kühlung: Gruppe A und Gruppe B) | 30 min |
| Experiment Tag 4 | Folgemessung 72 Stunden (Kühlung: Gruppe A und Gruppe B) | 30 min |
| | | |
| **Totaler Zeitaufwand pro ProbandIn** | | **180 min** |

**Rekrutierung**

Sobald die potenziellen ProbandInnen Interesse an der Untersuchung zeigen und im Labor in Landquart eintreffen, werden sie zum Studienleiter geschickt. Ist dieser zu diesem Zeitpunkt nicht anwesend oder verfügbar, werden die Interessierten gebeten, an einem anderen Tag zu erscheinen.

Der Studienleiter erläutert den Ablauf, die durchgeführten Tests, die Risiken sowie mögliche Unannehmlichkeiten. Zudem wird erklärt, dass die Teilnahme freiwillig ist und ein Rücktritt jederzeit, ohne Angabe von Gründen, möglich ist, ohne dass dies negative Auswirkungen auf die zukünftige Betreuung hat.

Anschließend erhalten die ProbandInnen die Probandeninformation, die die Untersuchung detailliert und verständlich beschreibt. Das Dokument enthält außerdem eine Checkliste, die ausgefüllt werden muss. Die Einwilligungserklärung wird ebenfalls bereitgestellt und muss vor Studienantritt vom Studienleiter unterschrieben zurückgegeben werden. Dieser prüft die ausgefüllte Checkliste und bestätigt die Einwilligung.

**Baseline measurements**

Die Raumtemperatur und die relative Luftfeuchtigkeit werden zu Beginn eines jeden Messtages ermittelt (Voltcraft MT52 Digitalmultimeter, Hirschau, Deutschland). Die Baseline-Messungen umfassen demografische Daten (Alter, Geschlecht, Größe). Zu den anthropometrischen Merkmalen zu Beginn der Untersuchung gehörten die Körpergröße (GPM Stadiometer, Zürich, Schweiz), die Körpermasse und die Schätzung des Körperfettanteils. Körpermasse und unterer Körperfettanteil wurden mit einer TANITA-TBF 611-Waage (Tokio, Japan) gemessen. Die Schätzung des prozentualen Anteils des unteren Körperfetts wurde gewählt, weil die Kältepackung am Oberschenkel angelegt wurde, was hauptsächlich die unteren Extremitäten betrifft.

Die Erholungsparameter (siehe genaues Messverfahren sowie primäre und sekundäre Endpunkte) werden bei jeder Messung in der gleichen Reihenfolge bewertet: (1) Entzündungswerte, (2) Muskelschwellung, (3) Muskelkater, (4) Maximale willkürliche isometerische Muskelkontraktion. Das Messverfahren und die für jedes spezifische Ergebnis verwendete Ausrüstung werden in Kapitel 3.2 beschrieben.

**Randomization**

Die Randomisierung der ProbandInnen erfolgt am Tag der Baseline-Messung durch Ziehung eines Loses. Es wird sichergestellt, dass für jede Interventionsgruppe (5 Lose pro Gruppe) und ProbandInnen die gleiche Anzahl an Losen vorhanden ist. Überschreitet die Probandanzahl einer Gruppe ein Drittel der Gesamtprobandanzahl, werden die Lose dieser Gruppe entfernt. Um die Gleichheit der Gruppen hinsichtlich Kovariaten wie Geschlecht zu gewährleisten, wird die Zusammensetzung der Gruppen überwacht. Sobald die Hälfte einer Gruppe aus männlichen oder weiblichen ProbandInnen besteht, werden die Lose für diese Gruppe für zukünftige ProbandInnen desselben Geschlechts entfernt.

Die Lose bestehen aus gefaltetem Papier, auf dem die Zahlen 1, 2 oder 3 stehen, und werden gemeinsam in einen Behälter gelegt. Das Ziehen der Lose erfolgt durch die ProbandInnen selbst. Nach der Ziehung übergeben die ProbandInnen das ungelesene Los an den Studienleiter, der sie einer Gruppe zuweist.

**Blinding**

Da in dieser Untersuchung Kälteapplikationen getestet werden, ist eine Blindierung nicht möglich.

**Muskelkaterprotokoll**

Um Muskelkater zu induzieren, wird ein einbeiniges Kniestrecker-Protokoll angewendet, welches bereits verwendet wurde (Ruas et al., 2022). Die Teilnehmer werden angewiesen, 6 Sätze ihr Knie zu strecken und zu beugen für 12 – 15 Wiederholungen, mit 2 Minuten Pause zwischen den Sätzen. Während der 2-minütigen Pause dürfen sich die Teilnehmer auf den Stuhl setzen. Die Ausführung des Kniestrecker-Protokolls wird von einem Prüfer visuell beobachtet. Verbale Korrekturen der Ausführung können vom Prüfer gegeben werden. Es wird jedoch keine verbale Ermutigung gegeben. Alle Trainingseinheiten werden am Vormittag durchgeführt. Kann ein Teilnehmer aufgrund extremer Ermüdung oder maximaler Anstrengung nicht alle Wiederholungen eines Satzes ausführen, kann der Satz abgebrochen und nach der Ruhezeit ein neuer Satz begonnen werden. Es müssen jedoch mindestens 12 Wiederholungen pro Satz durchgeführt werden. Andernfalls wird dies als Abbruch gewertet.

**Intervention**

Die Probanden in der Gruppe A (sofortige Kühlung) erhalten sofort nach Beendigung des Muskelkaterprotokolle die erste Kühlung auf die beiden Oberschenkel. Diese Kühlung wird in Rückenlage durchgeführt. Nach dieser Applikationen können sie das Labor verlassen und die Messung der Hauttemperatur wird gestoppt.

Die Gruppe B (verzögerte Kühlung) und Gruppe C (Kontrollgruppe) erhalten direkt nach dem Muskelkaterprotokoll noch keine Kühlung (Gruppe B) bzw. irgendeine Intervention (Gruppe C). Sie müssen jedoch ebenfalls noch 20 min in Rückenlage im Labor verbringen, bevor die Hauttemperatur-messung beendet wird.

**Follow-up measurements**

Die Folgemessungen werden für jede Messung in der gleichen Reihenfolge wie für die Baseline-Messung durchgeführt ((1) Entzündungsmarker, (2) Muskelschwellung, (3) Muskelkater, (4) Maximale willkürliche isometrische Kontraktion). Die Follow-up Messungen finden 24, 48, und 72 Stunden nach dem Muskelkaterprotokoll im Labor in Landquart statt.

Das Messverfahren und die für jedes spezifische Ergebnis verwendete Ausrüstung werden in Kapitel 3.2 beschrieben.

### 3.4 Withdrawal and discontinuation

Die Teilnehmer können jederzeit und ohne Angaben von Gründen aus der Untersuchung aussteigen. Die Teilnehmer werden aus der Untersuchung ausgeschlossen, wenn eine drohende Verletzung oder Krankheit eine Übung oder Kälteanwendung kontraindiziert.

## 4 STATISTICS AND METHODOLOGY

### 4.1. Statistical analysis plan and sample size calculation

**Stichprobenberechnung**

Vor Durchführung jeder Datenerhebung ist eine Fallzahlplanung und Fallzahlberechnung notwendig, um valide Ergebnisse zu erhalten. Bei klinischen Studien und Tierversuchen ist diese sogar zwingend vorgeschrieben und wird vor Erteilung der Zulassung genau überprüft (MDR, Anhang XV, Kapitel 1, Abschnitt 2.1, DIN EN ISO 14155:2021-05). Die postoperative Kryotherapie unter Verwendung von Eisbeuteln, Kühlung oder kontinuierlichen Kryotherapiegeräten reduzierte die Schmerzwerte auf der visuellen Analogskala und den Analgetika Verbrauch in etwa bei der Hälfte der Forschungsstudien, in denen diese Ergebnisse gegen ihre Kontrolle (keiner Kryotherapie) verglichen wurden. Der durchschnittliche Initiale VAS Score nach Schmerzinduktion variiert je nach Studie und liegt als Näherung bei einem

Ausgangswert von 4,73 ± 2,39. Eine Reduktion des VAS-Scores nach Kälte Applikation wurde mit durchschnittlich 47% angenommen. Nach Berücksichtigung der Dropout-Rate von 10% und Aufsplittung in 3 randomisierte Gruppe wird eine Patientenzahl von 15 Probanden pro Studienarm als statistisch angemessen betrachtet. Die Ergebnisse wurden mit der Software PASS 2023 Version 23.0.1 ermittelt.

Numerische Ergebnisse für Mehrarmige Tests für den Unterschied zwischen Behandlungs- und Kontrollmitteln unter der Annahme gleicher Varianz

| | |
|---|---|
| **Lösen für:** | Fallzahl |
| **Gruppenverteilung:** | gleich (Nc = N1 = N2 = ...) |
| **Test Typ:** | T-Test |
| **Hypothese:** | H0: δ = 0 vs. H1: δ ≠ 0 |
| **Anzahl an Gruppen:** | 3 |
| **Bonferroni Korrektur:** | keine (Divisor = 1) |

| Vergleich | Zielstärke | Wirklich | Fallzahl | Mittelwert $\mu$ i | Unterschied δi | Standardabweichung σ | Alpha |
|---|---|---|---|---|---|---|---|
| Kontrolle | | | 13 | 4,72 | | 2,1 | |
| vs. A | 0,8 | 0,83233 | 13 | 2,21 | -2,51 | 2,1 | 0,05 |
| vs. B | 0,8 | 0,91287 | 13 | 1,87 | -2,85 | 2,1 | 0,05 |
| Gesamt | n/a | n/a | 39 | n/a | n/a | n/a | n/a |

| | |
|---|---|
| **Vergleich** | Die Gruppe, die am Vergleich zwischen der in dieser Berichtszeile angezeigten Behandlung und Kontrolle beteiligt ist. Der Vergleich erfolgt anhand der Differenz. |
| **Teststärke** | Die gewünschte Teststärke. Die Teststärke ist die Wahrscheinlichkeit, eine falsche Nullhypothese für diesen Vergleich abzulehnen. Diese Teststärke bezieht sich nur auf den in dieser Zeile gezeigten Vergleich. |
| **Wirkl. TS** | Die Teststärke die wirklich erreicht wird. |
| **Ni** | Die Anzahl der Elemente in der i-ten Gruppe. Die Gesamtstichprobengrösse wird in der letzten Zeile der Spalte angezeigt. |
| **$\mu$ i** | Der Mittelwert der i-ten Gruppe, bei der die Teststärke berechnet wird. Die erste Zeile enthält $\mu$ c, den Mittelwert der Kontrollgruppe. |
| **δi** | Die Differenz zwischen dem i-ten Behandlungsmittel und dem Kontrollmittel ($\mu$ i – $\mu$ c), bei dem die Teststärke berechnet wird. |
| **σ** | Die Standardabweichung der Antworten innerhalb jeder Gruppe. |

| **Alpha** | Die Wahrscheinlichkeit, die Nullhypothese abzulehnen, dass der Kontrollmittelwert dem in dieser Zeile beschriebenen Behandlungsmittelwert entspricht. |
|---|---|

**Zusammenfassende Aussagen**
Ein paralleles 3-Gruppen-Design (mit einer Kontrollgruppe und 2 Behandlungsgruppen) wird verwendet, um zu testen, ob der Mittelwert für jede Behandlungsgruppe unterschiedlich ist aus dem Mittelwert der Kontrollgruppe (H0: $\delta = 0$ versus H1: $\delta \neq 0$, $\delta = \mu i - \mu c$). Die Hypothesen werden anhand von zwei zweiseitigen, zwei Stichproben umfassenden Methoden bewertet. Bonferroni-bereinigte t-Tests mit gleicher Varianz und einer gesamten (experimentellen) Typ-I-Fehlerrate ($\alpha$) von 0,05. Die gemeinsame Standardabweichung für alle Gruppen wird mit 2,1 angenommen. Der Mittelwert der Kontrollgruppe wird mit 4,72 angenommen. Die Behandlung bedeutet 2,21 und 1,87 mit jeweils mindestens 80 % Teststärke zu erkennen. Für den Test beträgt die (gleiche) Gruppenstichprobengrösse, die für jede der drei Gruppen (Kontrolle und Behandlungen) benötigt wird, 13 (insgesamt 39 Probanden). Die Gruppenstichprobengrössen wurden mit PASS 2023, Version 23.0.1 berechnet.
Stichprobengrösse mit Berücksichtigung der Dropoutrate

| Gruppe | Dropouts Abbruchquote | Fallzahl/Gruppe | Fallzahl mit Abbruchquote | Anzahl der Abbrechenden Teilnehmer |
|---|---|---|---|---|
| 1 - 3 | 10% | 13 | 15 | 2 |
| Gesamt | n/a | 39 | 45 | 6 |

Unter Berücksichtigung der Abbruchquote
Unter Berücksichtigung einer Abbrecherquote von 10 % sollte die Gruppengrössen in jedem Studienarm 15 Probanden betragen.
**Statistisches vorgehen, um die primären Endpunkte zu untersuchen**
Die statistische Analyse wird mittels IBM SPSS Statistics (Version 27, IBM Corp., Armonk, NY, USA) durchgeführt und das Signifikanzlevel auf $p < 0.05$ festgelegt.
Um die Zwischengruppenunterschiede feststellen zu können, wird eine wiederholte ANOVA Analyse durchgeführt mit dem Faktor 1: Zeit (Baseline, 24 Stunden, 48 Stunden, 72 Stunden) und dem Faktor 2: Intervention (Gruppe A, Gruppe B, Gruppe C) für die Erholungsparameter (Entzündungswerte, Muskelschwellung, Muskelkaterangaben, Maximale willkürliche isometrische Muskelkontraktion).
Die Untersuchung wird abgebrochen, wenn bei den Teilnehmern eine Unverträglichkeit mit der lokalen Kälteanwendung besteht. Die Teilnehmer der IC- und DC-Gruppe werden angewiesen, eine Kühlsitzung abzubrechen und sich umgehend an ihre Kontaktperson zu wenden, falls bei ihnen infolge der Kühlung Symptome wie starke Schmerzen/Beschwerden auftreten.

### 4.2. Handling of missing data and drop-outs

Fehlende Daten, verpasste Trainingseinheiten oder Abbrüche werden im CRF vermerkt. Daten von Patienten, die die Untersuchung abbrechen, werden erfasst und gespeichert. Drop-outs werden nicht durch die Rekrutierung neuer Teilnehmer ersetzt. Wird die Einwilligung zurückgezogen, werden die bereits erhobenen Daten aus wissenschaftlichen und Sicherheitsgründen in die Analyse einbezogen. Diese werden erst nach der Analyse anonymisiert (Art. 32 ClinO-MD).

# 5 SAFETY

## 5.1 Definition and Assessment of (Serious) Adverse Events and other safety related events

**Unerwünschtes Ereignis** (Adverse Event, AE) (Art. 2 Abs. 57 MDR)
Jedes ungünstige medizinische Ereignis, unbeabsichtigte Krankheit oder Verletzung oder jedes ungünstige klinische Anzeichen (einschließlich eines abnormen Laborbefunds) bei Probanden, Anwendern oder anderen Personen, unabhängig davon, ob es mit dem Medizinprodukt (MP) in Zusammenhang steht oder nicht.
Dazu zählen Ereignisse im Zusammenhang mit dem untersuchten MP oder dem Vergleichsprodukt sowie den damit verbundenen Verfahren. Für Anwender oder andere Personen beschränkt sich dies auf Ereignisse, die mit dem MP in Zusammenhang stehen.

**Schwerwiegendes unerwünschtes Ereignis** (Serious Adverse Event, SAE) (Art. 2 Abs. 58 MDR)
Ein unerwünschtes Ereignis, das zu einem der folgenden führte:

- (a) Tod,
- (b) schwerwiegende Verschlechterung der Gesundheit des Probanden, die zu einem der folgenden führte:
  - (i) lebensbedrohliche Krankheit oder Verletzung,
  - (ii) dauerhafte Beeinträchtigung einer Körperstruktur oder -funktion,
  - (iii) Krankenhausaufenthalt oder Verlängerung eines bestehenden Krankenhausaufenthalts,
  - (iv) medizinischer oder chirurgischer Eingriff zur Verhinderung einer lebensbedrohlichen Krankheit oder Verletzung oder einer dauerhaften Beeinträchtigung einer Körperstruktur oder -funktion,
  - (v) chronische Krankheit,
- (c) fetale Belastung, Tod des Fötus oder eine angeborene körperliche oder geistige Beeinträchtigung oder ein Geburtsfehler.

Hinweis: Geplante Krankenhausaufenthalte aufgrund eines vorbestehenden Zustands oder eines im Prüfplan (CIP) festgelegten Verfahrens ohne schwerwiegende Verschlechterung des Gesundheitszustands des Probanden gelten nicht als SAE.

**Mängel des Medizinprodukts** (Device Deficiency) (Art. 2 Abs. 59 MDR)
Unzulänglichkeiten eines Medizinprodukts im Zusammenhang mit Identität, Qualität, Haltbarkeit, Zuverlässigkeit, Sicherheit oder Leistung eines Prüfprodukts, einschließlich Fehlfunktionen, Anwenderfehlern und unzureichender Informationen des Herstellers.
Die Definition schließt Mängel sowohl des Prüfprodukts als auch des Vergleichsprodukts ein.

**Fehlfunktion** (Malfunction) (ISO14155)
Das Versagen eines Prüfprodukts, seine beabsichtigte Funktion gemäß der Gebrauchsanweisung oder des Prüfplans (CIP) zu erfüllen.

**Mängel des Medizinprodukts mit SAE-Potenzial** (Device Deficiency with Serious Adverse Event Potential)
(Art. 80 Abs. 1 Buchstabe c MDR; ISO14155)
Jede Produktmängel, die zu einem schwerwiegenden unerwünschten Ereignis hätte führen können, wenn keine geeigneten Maßnahmen ergriffen worden wären, keine Intervention erfolgt wäre oder die Umstände ungünstiger gewesen wären.

Unerwünschte Wirkung des Medizinprodukts (Adverse Device Effect, ADE) (ISO14155)
Ein unerwünschtes Ereignis, das möglicherweise, wahrscheinlich oder ursächlich mit der Verwendung eines Prüfprodukts oder der damit verbundenen Verfahren zusammenhängt.

Dazu zählen Ereignisse, die aus unzureichenden oder fehlerhaften Gebrauchsanweisungen, aus dem Einsatz, der Implantation, Installation oder dem Betrieb oder aus Fehlfunktionen des Prüfprodukts resultieren. Dies schließt auch Ereignisse durch Bedienungsfehler oder absichtlichen Fehlgebrauch ein.

**Schwerwiegende unerwünschte Wirkung des Medizinprodukts** (Serious Adverse Device Effect, SADE) (ISO14155)
Eine unerwünschte Wirkung des Medizinprodukts (ADE), die zu einer der Folgen eines schwerwiegenden unerwünschten Ereignisses geführt hat.

**Unerwartete schwerwiegende unerwünschte Wirkung des Medizinprodukts** (Unanticipated Serious Adverse Device Effect, USADE) (ISO14155)
Eine schwerwiegende unerwünschte Wirkung des Medizinprodukts (SADE), die in ihrer Art, Häufigkeit, Schwere oder ihrem Ergebnis in der aktuellen Version des Risikoberichts nicht identifiziert wurde.
Hinweis: Eine erwartete schwerwiegende unerwünschte Wirkung des Medizinprodukts (Anticipated SADE, ASADE) ist eine Wirkung, die in der Art, Häufigkeit, Schwere oder ihrem Ergebnis bereits im Risikobericht identifiziert wurde.

**Kausaler Zusammenhang von SAEs** (MDCG 2020-10/1)
Der kausale Zusammenhang mit dem Medizinprodukt oder den Verfahren der Untersuchung wird durch den Hauptprüfer und den Sponsor wie folgt bewertet:

- Nicht verbunden: Der Zusammenhang mit dem Produkt oder den Verfahren kann ausgeschlossen werden.
- Möglich: Der Zusammenhang mit der Verwendung des Prüfprodukts ist schwach, aber nicht vollständig auszuschließen. Alternative Ursachen sind ebenfalls möglich. Fälle, bei denen die Bewertung nicht möglich ist oder keine Informationen vorliegen, werden ebenfalls als möglich eingestuft.
- Wahrscheinlich: Der Zusammenhang mit der Verwendung des Prüfprodukts erscheint relevant und/oder das Ereignis kann nicht sinnvoll durch eine andere Ursache erklärt werden.
- Kausaler Zusammenhang: Das schwerwiegende Ereignis ist zweifelsfrei mit dem Prüfprodukt oder den Verfahren verbunden.

### 5.2 Documentation and reporting in Medical Device Category A clinical investigations

Gerätemängel (DD) und alle unerwünschten Ereignisse (AE), einschließlich aller schwerwiegenden unerwünschten Ereignisse (SAE), werden während des gesamten Untersuchungszeitraums, d. h. vom Zeitpunkt der Einwilligung des Patienten bis zum letzten CIP-spezifischen Verfahren einschließlich einer Sicherheitsnachbeobachtungsphase, erfasst, vollständig untersucht und im Quelldokument sowie in den entsprechenden Case Report Forms (CRF) dokumentiert.

- **Dokumentation von AEs (einschließlich SAEs)** durch den Prüfer umfasst Diagnose oder Symptome, Beginn- und Enddatum des Ereignisses, Behandlung des Ereignisses, Lösung des Ereignisses, Bewertung der Schwere und des Kausalzusammenhangs mit dem Medizinprodukt und/oder dem Untersuchungsverfahren (Art. 32 ClinO-MD, ISO14155).
- **Dokumentation von DDs** durch den Prüfer umfasst die Beschreibung des Ereignisses, das Startdatum, Informationen zum Untersuchungsgerät, ergriffene Maßnahmen in Bezug auf das Untersuchungsgerät und die Feststellung, ob der DD zu einem AE geführt hat. Der Sponsor prüft alle DDs und entscheidet sowie dokumentiert schriftlich, ob sie zu einem SAE hätten führen können (DD mit SADE-Potenzial) (Art. 32 ClinO-MD, ISO14155).

**Reporting of SAEs (**Art. 32 ClinO-MD**)**
Alle SUEs werden dokumentiert und unverzüglich (innerhalb von maximal 24 Stunden) an den Sponsor/Investigator der Untersuchung gemeldet.
Wenn nicht ausgeschlossen werden kann, dass das in der Schweiz aufgetretene SUE auf die untersuchte Intervention zurückzuführen ist, meldet der Prüfer es innerhalb von 15 Tagen über BASEC an die Ethikkommission.

**Follow up of (Serious) Adverse Events**
Für die betroffenen Probanden wird ein Nachsorgeplan erstellt, der sicherstellt, dass sie eine angemessene medizinische Versorgung und Unterstützung erhalten. Die Kommunikation mit den betroffenen Probanden wird aufrechterhalten, um ihre Genesung zu überwachen.

**Notification of safety and protective measures (**Art. 32 ClinO-MD**)**
Müssen während der Durchführung der Untersuchung unmittelbare Sicherheits- und Schutzmaßnahmen ergriffen werden, so unterrichtet der Prüfer die Ethikkommission innerhalb von 7 Tagen über diese Maßnahmen und die Umstände, die sie erforderlich machen.

### 5.2.1 Foreseeable adverse events and anticipated adverse device effects

Vorhersehbare AEs während der Anwendung der MD.

| **Art des Ereignisses** | **Beschreibung** | **Maßnahmen zur Minderung oder Behandlung** | **Wahrscheinliche Inzidenz** |
|---|---|---|---|
| Hautreizungen oder Rötungen | Lokalisierte Erytheme oder Reizungen an der Anwendungsstelle durch Druck oder Kälte. | Schutzmaterialien verwenden; bei Bedarf beruhigende Cremes oder Gele auftragen. | Mittel |
| Schmerzen oder Unwohlsein | Vorübergehende Schmerzen oder Unwohlsein während oder nach der Anwendung durch die Kühlung. | Teilnehmer auffordern, Schmerzen zu melden. | Gering |
| Verminderte lokale Sensibilität | Vorübergehende Taubheit oder verminderte Sensibilität durch Kälteeinwirkung. | Teilnehmer über diese Möglichkeit informieren; längere Anwendung vermeiden, um Komplikationen vorzubeugen. | Mittel |
| Leichte Schwellung oder Ödeme | Lokalisierte Schwellungen durch verlängerte Kühlung oder unsachgemäße Anwendung. | Anwendungszeit überwachen; betroffenen Bereich hochlagern; Anwendungsdauer bei nachfolgenden Anwendungen reduzieren. | Gering |

Es werden keine SAEs erwartet.

### 5.2.2 Reporting of Safety related events

**Reporting to the Sponsor:**
Alle schwerwiegenden unerwünschten Ereignisse (SAEs), Gerätemängel (DDs) mit Potenzial für SAEs und Gesundheitsrisiken, die Maßnahmen erfordern, werden vom Prüfer (oder autorisierten Vertreter) unverzüglich an den Sponsor gemeldet, sobald er von dem Ereignis Kenntnis erlangt hat. DDs werden dahingehend bewertet, ob sie zu einem SAE führen könnten.

**Pregnancies**
Schwangere oder stillende Frauen sind von der Teilnahme an der Untersuchung ausgeschlossen. Teilnehmerinnen, die während der Untersuchung schwanger werden, müssen dies dem Studienleiter unverzüglich mitteilen und dürfen nicht weiter an der Untersuchung teilnehmen. Die Untersuchung stellt jedoch kein Risiko für Schwangere dar.

**Reporting to the Competent Ethics Committee:**
Der Sponsor meldet der Ethikkommission unverzüglich jedes SAE, bei dem ein kausaler Zusammenhang zwischen dem Ereignis und dem Testverfahren der klinischen Prüfung festgestellt wurde (Art. 33 ClinO-MD).
Um eine rasche Benachrichtigung sicherzustellen, kann der Sponsor zunächst eine unvollständige Meldung einreichen.
Falls während der Durchführung der Untersuchung Sicherheits- oder Gesundheitsrisiken auftreten, die sofortige Maßnahmen erfordern, informiert der Sponsor die Ethikkommission innerhalb von 2 Tagen über diese Maßnahmen und die Umstände, die sie erforderlich gemacht haben (Art. 34 ClinO-MD).

**Periodic safety reporting (Art. 35 ClinO-MD):**
Einmal jährlich reicht der Sponsor bei der Ethikkommission eine Liste der SAEs und DDs ein und legt einen Bericht über deren Schwere, den kausalen Zusammenhang mit dem Gerät und der Intervention sowie über die Sicherheit der Teilnehmer vor. Der Sponsor informiert die Ethikkommission jährlich über den allgemeinen Fortschritt der klinischen Untersuchung.

**Materiovigilance reporting to Swissmedic:**
Der Sponsor ist dafür verantwortlich, sicherzustellen, dass Swissmedic über schwerwiegende Vorfälle gemäß Art. 66 MedDO informiert wird.
Materiovigilance-Berichte werden nicht an die Ethikkommission gesendet.
Falls der Sponsor nicht der Hersteller des untersuchten Geräts oder der Schweizer Vertreter des Herstellers ist:

- Im Fall von Vorfällen muss überprüft werden, ob das Ereignis gemäß Art. 66 Abs. 4 MedDO der Meldepflicht unterliegt (unter Verwendung der Anleitung MU680_20_008e_WL).
- Der Sponsor muss sicherstellen, dass meldepflichtige Vorfälle mit dem Formular MU680_20_015d_FO an Swissmedic gesendet werden (materiovigilance@swissmedic.ch).
- Nutzer sind gesetzlich verpflichtet, die Lieferanten der Geräte über schwerwiegende Vorfälle zu informieren (Art. 66 Abs. 4 MedDO).

## 5.3 Radiation

In dieser Untersuchung ist keine Strahlung enthalten.

## 5.4 Amendments (Art. 15 ClinO-MD)

Wesentliche Änderungen des Studienaufbaus, der Organisation, des Protokolls und der zugehörigen Studiendokumente werden gemäss Art. 15 ClinO-MD der Ethik-Kommission vor der

Durchführung zur Genehmigung vorgelegt. Zusätzlich wird eine Liste der nicht-wesentlichen Änderungen jährlich der EK gemeldet zusammen mit dem ASR oder dem *general study progress report*.

### 5.5 Notification and reporting upon completion, discontinuation or interruption of the Investigation

Nach dem regulären Abschluss der Untersuchung wird die Ethikkommission gemäß Artikel 38 der ClinO-MD innerhalb von 30 Tagen über BASEC informiert.
Der Sponsor/Prüfer kann die Untersuchung unter bestimmten Umständen vorzeitig beenden, z. B. bei:

- Ethischen Bedenken,
- Unzureichender Teilnehmerrekrutierung,
- Zweifeln an der Sicherheit der Teilnehmenden oder bei Gefährdung der Teilnehmenden (z. B. wenn die Nutzen-Risiko-Bewertung nicht mehr positiv ausfällt),
- Änderungen in der anerkannten klinischen Praxis, die die Fortsetzung der Untersuchung als nicht mehr sinnvoll erscheinen lassen,
- Frühen Hinweisen auf Schaden oder Nutzen der experimentellen Intervention.

Im Fall eines vorzeitigen Studienabbruchs oder einer Studienunterbrechung wird die Ethikkommission gemäß Artikel 38 der ClinO-MD innerhalb von 15 Tagen über BASEC informiert.

Innerhalb von 12 Monaten nach Abschluss oder Abbruch der Untersuchung wird der Ethikkommission über BASEC ein Abschlussbericht vorgelegt, sofern im Prüfplan keine längere Frist vorgesehen ist (Artikel 38 ClinO-MD).

### 5.6 Insurance

Es besteht eine Betriebshaftpflichtversicherung bei der Basler Versicherung für die Thim van der Laan AG in Landquart, in welcher die Untersuchung durchgeführt wird.

## 6 FURTHER ASPECTS

### 6.1 Overall ethical considerations

Die Untersuchung wird an gesunden Freiwilligen durchgeführt. Die Teilnehmer können jederzeit ohne Konsequenzen aus der Untersuchung aussteigen, wie in der Einwilligungserklärung angegeben. Die Stichprobe gesunder Teilnehmer im Alter zwischen 18 und 30 Jahren spiegelt eine Bevölkerung wider, die sich regelmäßig körperlich betätigt, so dass die Ergebnisse auf die allgemeine Bevölkerung übertragbar sind. Um die Sicherheit der Teilnehmer in den Vordergrund zu stellen, wurden gefährdete Bevölkerungsgruppen wie Schwangere und Personen mit bestimmten Gesundheitszuständen ausgeschlossen, um mögliche unerwünschte Reaktionen zu minimieren. Die Ergebnisse dieser Untersuchung können wertvolle Erkenntnisse über die Mechanismen und die Anwendung von Erholungsstrategien nach dem Sport liefern. Die Verwendung eines randomisierten kontrollierten Studiendesigns ist wissenschaftlich rigoros und minimiert Verzerrungen. Durch die Berücksichtigung von Kovariaten (z. B. Geschlecht) bei der Randomisierung erhöht die Untersuchung die interne Validität und reduziert das Potenzial für Störvariablen. In der Einwilligungserklärung wird ausdrücklich festgehalten, dass die Teilnehmer über persönliche Befunde und zufällige Ergebnisse, die für ihre Gesundheit relevant sind (z. B. abnorme Blutwerte), informiert werden.

### 6.2 Risk-benefit assessment

Mit der Teilnahme an dieser Untersuchung sind keine gesundheitlichen Risiken verbunden. Die in dieser Untersuchung verwendeten CE-zertifizierten Kältepackungen (Axanova Cold Hot Pearls Maxi Pack, Axanova AG) wurden speziell entwickelt, um einen sicheren Kühleffekt auf das betroffene Muskelgewebe auszuüben. Das Produkt wird entsprechend der Gebrauchsanweisung verwendet. Wie in der Einwilligungserklärung angegeben, wird in der Gebrauchsanweisung der CE-zertifizierten Kältepackungen (Axanova Cold Hot Pearls Maxi Pack, Axanova AG) auf die Möglichkeit einer Überkühlung und der anschließenden Entwicklung lokaler Erfrierungen durch lokale Kälteanwendung hingewiesen. Durch ausdrückliche Anweisungen zur Anwendung, Dauer und Häufigkeit trägt das Protokoll dazu bei, dass die Teilnehmer die Kühlakkus korrekt anwenden, wodurch das Risiko unerwünschter Wirkungen minimiert und die beabsichtigte angemessene Kühlwirkung auf das Muskelgewebe optimiert wird.

Wie in der Einwilligungserklärung angegeben, müssen die Teilnehmer damit rechnen, dass sie aufgrund der körperlichen Anstrengung durch das Kniestrecker-Protokoll und die Messung des maximalen isometrischen Kontraktionslevel, die mit einer mittleren bis hohen Intensität verbunden sind, ermüden und Muskelkater bekommen. Diese Beschwerden werden jedoch nicht als signifikantes Gesundheitsrisiko angesehen. Das Studiendesign zielt darauf ab, eine sichere und kontrollierte Übungsumgebung mit minimalem Verletzungspotenzial zu gewährleisten.

Venöse Blutproben wurden von einem geschulten Spezialisten aus einer Cubitalvene entnommen. Wie in der Einverständniserklärung zur Blutentnahme angegeben, besteht jedoch die Möglichkeit, dass an der Einstichstelle Blutergüsse, Blutungen oder Schwellungen auftreten.

Die Teilnahme an der Untersuchung bringt keinen direkten Nutzen für die Teilnehmer mit sich. Auch wenn es keinen direkten persönlichen Nutzen gibt, hat die Beteiligung an dieser Untersuchung das Potenzial, wertvolle Daten für die wissenschaftliche Gemeinschaft zu liefern. Die Ergebnisse könnten künftigen Sportlern, Personen, die sich körperlich betätigen, und Patienten zugutekommen, indem sie Aufschluss über die besten Verfahren zur Erholung nach dem Sport geben.

## 7. QUALITY CONTROL AND DATA PROTECTION

### 7.1 Quality measures

Das Monitoring wird von Dr. Biomed. Ing. Ursula Hohenauer-Küng von der Institution THIM - Die internationale Hochschule für Physiotherapie, Weststrasse 8, 73002 Landquart durchgeführt.

Dr. biomed. Ing. Ursula Hohenauer-Küng gewährleistet in ihre Funktion als Monitor der Untersuchung, dass die Untersuchung korrekt durchgeführt und dokumentiert wird. Eine detaillierte Beschreibung ihrer Tätigkeiten als Monitor wird unten im Punk 9 aufgelistet. Darüber ist gewährleistet, dass Dr. Biomed. Ing. Ursula Hohenauer-Küng unabhängig vom Studienteam ist und steht nicht beim Prüfer der Untersuchung im Anstellungsverhältnis.

Zur Qualitätssicherung können der Sponsor, die Ethik-Kommission oder ein unabhängiger Studienmonitor die Forschungsstätten besuchen. Bei solchen Gelegenheiten wird direkter Zugang zu den Quelldaten und allen studienbezogenen Dateien gewährt. Alle beteiligten Parteien behandeln die Probandendaten streng vertraulich.

### 7.2 Data recording and source data

Die Antworten auf die Checkliste, die demografischen und anthropometrischen Daten (Geschlecht, Alter, Größe, Gewicht, geschätzter unterer Körperfettanteil), die Raumbedingungen (Temperatur, relative Luftfeuchtigkeit), Muskelkater und maximale isometrische Kontraktionskraft werden manuell im CRF erfasst. Im Anschluss werden die Daten in Microsoft Excel übertragen auf den Forschungscomputer des Forschungslabors. Aktivierung der Option „Track Changes“ wird im Excel-Datei zur Sicherstellung des Datenschutzes und der Datensicherheit gewährleistet. Zusätzlich wird die Excel-Datei passwortgeschützt und Back-ups werden regelmässig nach Messungen durchgeführt.
Die Studienteilnehmer, die die Applikation zu Hause durchführen und dokumentieren, laden die Bilder direkt über einen sicheren, passwortgeschützten Link in einen dafür vorgesehenen Ordner auf Microsoft OneDrive for Business hoch. Dabei wird Azure Information Protection (AIP) eingesetzt, um eine sichere Übertragung (mit TLS/SSL-Verschlüsselung) sowie eine sichere Speicherung der Daten zu gewährleisten. Die hochgeladenen Bilder sind ausschließlich für autorisierte Mitglieder des Studienteams zugänglich, und die Speicherung erfolgt in Übereinstimmung mit den geltenden Datenschutzvorschriften. Nach Abschluss der Untersuchung oder nach Ablauf der Aufbewahrungsfrist werden die Bilder sicher und vollständig gelöscht.
Die Blutsenkgeschwindigkeit wird vor Ort im Forschungslabor anhand der Westergren Methode bestimmt. Im Anschluss werden die Blutproben zerstört. Die restlichen Parameter werden von einem externen Labor (Dr. Riesch, Buchs) bestimmt. Nach der Blutabnahme werden die Proben im Verlauf desselben Tages von einem Blutkurier abgeholt und in das medizinische Labor gebracht, wo die standardisierten Analysen durchgeführt werden. Die Kreatin-kinase -Werte werden mit der Ultraviolett-Methode und die C-reaktives Protein-Werte mit der turbidimetrischen Methode bestimmt. Die Blutproben werden vom externen Labor vernichtet, und die Berichte werden uns zugesendet. Wir bewahren diese Berichte für die Dauer von 10 Jahren vertraulich auf. Das externe Labor erhält nur den nummerischen Code der Teilnehmer (z.B. 001, 002, 003, etc.). Es können keinerlei Rückschlüsse vom externen Labor auf die ProbandInnen getroffen werden.
Die Bilder für die Untersuchung der Muskelschwellung werden auf einem Ultraschallgerät gespeichert. Es werden nur der nummerische Code im Ultraschallgerät hinterlegt und keine personengebundenen Daten. Es ist nicht möglich, anhand des nummerischen Codes, Rückschlüsse auf die ProbandInnen zu ziehen. Das Ultraschallgerät ist Eigentum des Forschungslabors und wird nur von Forschungsmitarbeitern bedient.
Der Schlüssel für den Code befindet sich nur beim Studientleiter dieser Untersuchung. Nur der Studienleiter kann den Code entschlüsseln.

### 7.3 Confidentiality and coding

Studien- und Teilnehmerdaten werden mit äußerster Diskretion behandelt und sind nur autorisiertem Personal zugänglich, das die Daten zur Erfüllung seiner Aufgaben im Rahmen der Untersuchung benötigt. Auf den CRFs und anderen studienspezifischen Dokumenten werden die Teilnehmer nur durch eine nummerische Teilnehmernummer identifiziert. Es werden keine persönlichen Daten präsentiert oder publiziert. Die unterzeichnete Einverständniserklärung sowei die ausgefüllte Checkliste werden im Original als Studiendokument in einem abgeschlossenen Aktenschrank aufbewahrt.

## 7.4 Retention and destruction of Investigation data and biological material

Alle Studiendaten werden nach Studienende oder vorzeitigem Abbruch der Untersuchung für 20 Jahre archiviert. Nach Ablauf dieser Frist werden alle Daten gemäß den geltenden Datenschutzvorschriften und den Richtlinien des Studienprotokolls sicher vernichtet. Dies betrifft sowohl die elektronischen Daten als auch alle biologischen Proben, die im Rahmen der Untersuchung gesammelt wurden. Die Vernichtung erfolgt auf sichere Weise, etwa durch Verschlüsselung und vollständige Löschung der elektronischen Daten und durch die physische Zerstörung biologischer Materialien, um jede Möglichkeit der Rekonstruktion oder der Identifikation von Probanden auszuschließen. Sollte eine längere Aufbewahrung notwendig sein, etwa aus regulatorischen oder wissenschaftlichen Gründen, wird dies zuvor mit den zuständigen Ethikkommissionen und Behörden abgestimmt.

# 8 MONITORING AND REGISTRATION

Das Monitoring wird von Dr. Ursula Hohenauer-Küng von der Institution THIM - Die internationale Hochschule für Physiotherapie, Weststrasse 8, 73002 Landquart durchgeführt. Frau Dr. biomed. Ing. Ursula Hohenauer-Küng stellt sicher, dass die Untersuchung ordnungsgemäß durchgeführt und dokumentiert wird, indem sie die folgenden Tätigkeiten ausführt, sofern sie für die Untersuchung relevant und notwendig sind:

- Überprüfung, ob der Prüfer über angemessene Qualifikationen und Ressourcen verfügt und diese während der gesamten Studiendauer beibehält, ob die Einrichtungen, einschließlich der Labors, der Ausrüstung und des Personals, für die sichere und ordnungsgemäße Durchführung der Untersuchung geeignet sind und während der gesamten Studiendauer beibehalten werden.
- Überprüfung der Studieneingriffe
- Sicherstellung, dass die Bedingungen für die Interventionen akzeptabel sind.
- Überprüfung, dass die Studieninterventionen nur den Probanden zur Verfügung gestellt werden, die dafür in Frage kommen.
- Sicherstellung, dass den Prüfungsteilnehmern die erforderlichen Anweisungen zu den Studieneingriffen erteilt werden.
- Überprüfung, ob der Prüfer den genehmigten Prüfplan und ggf. alle genehmigten Änderungen einhält.
- Überprüfung, ob vor der Teilnahme eines jeden Prüfungsteilnehmers an der Untersuchung eine schriftliche Einwilligung nach Aufklärung eingeholt wurde.
- Sicherstellung, dass der Prüfer und das Prüfpersonal des Prüfers angemessen über die Prüfung informiert sind.
- Überprüfung, ob der Prüfer nur in Frage kommende Prüfungsteilnehmer rekrutiert.
- Berichterstattung über die Probandenrekrutierungsrate.
- Überprüfung, ob der Prüfer alle erforderlichen Berichte, Meldungen, Anträge und Einreichungen vorlegt und ob diese Dokumente korrekt, vollständig, rechtzeitig, lesbar und datiert sind und die Prüfung identifizieren.
- Überprüfung, ob unerwünschte Ereignisse, Begleitmedikationen und interkurrente Erkrankungen gemäß dem Prüfplan auf den CRFs gemeldet werden.
- Sicherstellung, dass nicht wahrgenommene Besuche der Prüfungsteilnehmer, nicht durchgeführte Tests und Untersuchungen in den Prüfbögen eindeutig als solche ausgewiesen werden.
- Alle Rücktritte und Abbrüche von eingeschriebenen Probanden aus der Untersuchung werden auf den CRFs gemeldet und erläutert.

Source Data Verification (SDV) wird im Rahmen des Monitorings ebenfalls durchgeführt, um die Genauigkeit und Integrität der Studiendaten sicherzustellen. Dies umfasst die folgenden spezifischen Tätigkeiten:

- Überprüfung der Quellen und Originaldaten: Es wird überprüft, dass alle in den CRFs erfassten Daten mit den originalen, primären Datenquellen übereinstimmen, wie z. B. Teilnehmerdaten, Laborberichten und Notizen von Untersuchungen.
- Kontrolle der Dokumentation der Studienteilnehmer: Überprüfung, dass alle relevanten demografischen und medizinischen Daten (z. B. Alter, Geschlecht, Anamnese, Testergebnisse) korrekt erfasst sind und mit den originalen Aufzeichnungen übereinstimmen.
- Verifizierung der Einwilligungserklärungen: Sicherstellung, dass für jeden Teilnehmer eine schriftliche und informierte Einwilligung vor Studienbeginn vorliegt.
- Überprüfung von unerwünschten Ereignissen (AE): Bestätigung, dass alle unerwünschten Ereignisse und Begleitmedikationen korrekt und gemäß dem Prüfplan dokumentiert sind.
- Kontrolle der Studieninterventionen: Verifizierung, dass nur geeignete Teilnehmer die Studieninterventionen erhalten haben und dass alle Interventionsdaten ordnungsgemäß aufgezeichnet wurden.
- Prüfung der Abbrüche und Nichtwahrnehmungen: Überprüfung, dass alle Abbrüche von Studienbesuchen und Nichtwahrnehmungen ordnungsgemäß in den CRFs vermerkt und erklärt wurden.

Diese Maßnahmen gewährleisten, dass die Untersuchung den höchsten Standards in Bezug auf Datenintegrität und -sicherheit entspricht und dass alle verzeichneten Daten korrekt und verlässlich sind.

## 9. FUNDING / PUBLICATION / DECLARATION OF INTEREST

Diese Untersuchung wird von der Thim van der Laan AG in Landquart finanziert. Die Prüfer sind von diesem Institut angestellt und werden für die Mitarbeit an dieser Untersuchung entsprechend ihren Arbeitsverträgen entlohnt. Es wird keine weitere finanzielle Unterstützung benötigt.
Der Sponsor trägt die Zusammenfassung der Studienergebnisse gemäß ClinO Art. 65a innerhalb eines Jahres nach Abschluss oder Abbruch der Untersuchung in ein öffentliches Register ein und veröffentlicht sie. Eine Unterbrechung von mehr als zwei Jahren wird als Abbruch der Untersuchung gewertet. Für die Veröffentlichung im öffentlichen Register sorgt der Sponsor zudem dafür, dass eine leicht verständliche Zusammenfassung der Studienergebnisse innerhalb eines Jahres nach Abschluss oder Abbruch der Untersuchung in BASEC eingetragen wird. Der Eintrag erfolgt mindestens in der Landessprache der Schweiz, in der die Studienteilnehmer rekrutiert wurden.

## 10. REFERENCES

Bleakley, C., McDonough, S., Gardner, E., Baxter, G. D., Hopkins, J. T., & Davison, G. W. (2012). Cold-water immersion (cryotherapy) for preventing and treating muscle soreness after exercise. *Cochrane Database Syst Rev, 2*, CD008262. doi:10.1002/14651858.CD008262.pub2

Hohenauer, E., Taeymans, J., Baeyens, J. P., Clarys, P., & Clijsen, R. (2015). The Effect of Post-Exercise Cryotherapy on Recovery Characteristics: A Systematic Review and Meta-Analysis. *PLoS One, 10*(9), e0139028. doi:10.1371/journal.pone.0139028

Hubbard, T. J., & Denegar, C. R. (2004). Does Cryotherapy Improve Outcomes With Soft Tissue Injury? *J Athl Train, 39*(3), 278-279.

Ostrowski, J., Purchio, A., Beck, M., Leisinger, J., Tucker, M., & Hurst, S. (2018). Examination of Intramuscular and Skin Temperature Decreases Produced by the PowerPlay Intermittent Compression Cryotherapy. *J Sport Rehabil, 27*(3), 244-248. doi:10.1123/jsr.2016-0244

Ruas, C., Latella, C., Taylor, J., Gregory Haff, G., Nosaka, K. (2022). Comparison between eccentric-only and coupled concentric-eccentric contractions for neuromuscular fatigue and muscle damage. Med Sci Sports Exerc, 1;54(10):1635-1646. Doi: 10.1249/MSS.0000000000002959

Chow, S.C., Shao, J., Wang, H., and Lokhnygina, Y. 2018. Sample Size Calculations in Clinical Research, 3rd Edition. Chapman & Hall/CRC. Boca Raton, FL. Pages 86-88.

Machin, D., Campbell, M.J., Tan, S.B, and Tan, S.H. 2018. Sample Sizes for Clinical, Laboratory, and Epidemiology Studies, 4th Edition. Wiley Blackwell.

Julious, Steven A. 2004. 'Tutorial in Biostatistics. Sample sizes for clinical trials with Normal data.' Statistics in Medicine, 23:1921-1986.

Zar, Jerrold H. 1984. Biostatistical Analysis (Second Edition). Prentice-Hall. Englewood Cliffs, New Jersey.
